# A continuous morphological approach to study the evolution of pollen in a phylogenetic context: An example with the order Myrtales

**DOI:** 10.1371/journal.pone.0187228

**Published:** 2017-12-06

**Authors:** Ricardo Kriebel, Mohammad Khabbazian, Kenneth J. Sytsma

**Affiliations:** 1 Department of Botany, University of Wisconsin, Madison, Wisconsin, United States of America; 2 Department of Statistics, Columbia University, New York, New York, United States of America; Austrian Federal Research Centre for Forests BFW, AUSTRIA

## Abstract

The study of pollen morphology has historically allowed evolutionary biologists to assess phylogenetic relationships among Angiosperms, as well as to better understand the fossil record. During this process, pollen has mainly been studied by discretizing some of its main characteristics such as size, shape, and exine ornamentation. One large plant clade in which pollen has been used this way for phylogenetic inference and character mapping is the order Myrtales, composed by the small families Alzateaceae, Crypteroniaceae, and Penaeaceae (collectively the “CAP clade”), as well as the large families Combretaceae, Lythraceae, Melastomataceae, Myrtaceae, Onagraceae and Vochysiaceae. In this study, we present a novel way to study pollen evolution by using quantitative size and shape variables. We use morphometric and morphospace methods to evaluate pollen change in the order Myrtales using a time-calibrated, supermatrix phylogeny. We then test for conservatism, divergence, and morphological convergence of pollen and for correlation between the latitudinal gradient and pollen size and shape. To obtain an estimate of shape, Myrtales pollen images were extracted from the literature, and their outlines analyzed using elliptic Fourier methods. Shape and size variables were then analyzed in a phylogenetic framework under an Ornstein-Uhlenbeck process to test for shifts in size and shape during the evolutionary history of Myrtales. Few shifts in Myrtales pollen morphology were found which indicates morphological conservatism. Heterocolpate, small pollen is ancestral with largest pollen in Onagraceae. Convergent shifts in shape but not size occurred in Myrtaceae and Onagraceae and are correlated to shifts in latitude and biogeography. A quantitative approach was applied for the first time to examine pollen evolution across a large time scale. Using phylogenetic based morphometrics and an OU process, hypotheses of pollen size and shape were tested across Myrtales. Convergent pollen shifts and position in the latitudinal gradient support the selective role of harmomegathy, the mechanism by which pollen grains accommodate their volume in response to water loss.

## Introduction

Recent and continuing intellectual and computational advancements have facilitated the integration of phylogenetics, ecology, character evolution, biogeography, and rates of evolution, enabling evolutionary biologists to rigorously test hypotheses in ways not possible even a decade ago [1–5]. These advances are now impacting the manner in which morphological characters are being used in a phylogenetic context. The use of continuous characters in a phylogenetic framework has been a contentious, conceptually difficult topic for systematists due to potential lack of objectivity in character scoring [6, 7]. The recent use of geometric morphometrics to describe quantitative variables of an individual’s character(s), as its ‘morphospace’, in combination with explicit phylogenetic methods [5, 8–16] has provided one solution to this problem.

The evolution and systematic utility of pollen shape and size within seed plants have been studied by quantifying pollen grains with traditional morphometric methods that take into account linear measurements and/or meristic variables [17–21]. Pollen evolution has been studied in a phylogenetic context in just over a dozen cases [22–28]. These studies for the most part have reconstructed the ancestral states of pollen characters onto molecular phylogenies as discrete traits, or as continuous traits that were binned under certain criteria such as the gap weighting method implemented in the program MorphoCode [6, 29]. In the case of shape, it is usually discretized in categories including oblate, subspheroidal, or prolate [25, 30]. The evolution of pollen shape and size may be influenced by ecological factors such as shifts in mode of pollination [31] and by water availability. For example, harmomegathy or change in pollen volume to accommodate changes in water availability [32, 33] may be important in pollen evolution within clades that have experienced repeated biome shifts [1]. Geometric morphometric tools are just beginning to be used with pollen. These include landmark based analyses [34] and elliptic Fourier analyses (EFA) [35–37] of pollen shape outlines [38].

We present here, using a recent phylogenetic framework for the angiosperm order Myrtales [39], the largest study of pollen shape and size undertaken with these new approaches. The order Myrtales is one of the largest in the Angiosperms with around 12,000 species and is estimated to have diverged from Geraniales about 124 million years ago either in Africa or South America during the early stages of the break up of Gondwana [39]. Phylogenetic relationships within the order are for the most part well established [39–41], and currently nine families are recognized within Myrtales: Alzateaceae, Combretaceae, Crypteroniaceae, Lythraceae, Melastomataceae, Myrtaceae, Onagraceae, Penaeaceae, and Vochysiaceae [42]. Species vary widely in habit and life history, including annual herbs to large trees. Melastomataceae and Myrtaceae represent some of the most common understory shrub and tree species in the world’s tropical forests, whereas Onagraceae are a common element of temperate habitats and deserts. The evolution of floral form in Myrtales is intimately connected with pollinator diversity. In some cases a large clade is almost totally restricted in its pollinators such as the Melastomataceae, in which most of its about 5,500 species are pollinated by female bees that buzz the flowers for the pollen reward [43]. In other cases like Myrtaceae, complex mechanisms of secondary pollen presentation have evolved [44, 45]. Documented pollinators within the order are diverse and include beetles, thrips, flies, bees, hawkmoths, hummingbirds, passerine birds, bats, rodents, and small marsupials [43, 46–49]. By far the vast majority of species within the order appear to be pollinated by bees [43, 48].

The accompanying pollen diversity to this remarkable ecological and floral diversity (summarized comprehensively by [50]), not surprisingly, has been utilized in phylogenetic analyses of the Myrtales [50–54]. A pioneering morphological cladistic study included five binary pollen characters: presence or absence of syncolporate pollen, the shape of the pollen in equatorial view (“more or less oblate” or not), absence or presence of viscin threads, and the absence or presence of both subsidiary colpi and an “onagraceous exine” [53]. The potential of these characters as diagnostic for different families was demonstrated by mapping syncolporate pollen and pollen shape onto a molecular phylogeny [55]. More recently, fossil pollen in Myrtales, especially in the Lythraceae, Myrtaceae and Onagraceae, have been used to time calibrate molecular phylogenies [27, 39–41, 56, 57]. Onagraceae fossil pollen is particularly easy to identify due to the presence of viscin threads [58–60]. In other cases fossil pollen grains have been assigned with doubt, such as those of *Heterocolpites palaeocenica*, which were tentatively placed in the family Melastomataceae [61], whereas other fossil grains resembling *Heterocolpites* have been suggested to belong to the Combretaceae [52].

Here we use elliptic Fourier analysis to quantify shape variation from two-dimensional images of equatorial and polar views of pollen grains from almost 600 species of Myrtales to address specific hypotheses of pollen evolution using an expanded phylogenetic framework of the order. The extensive and descriptive documentation of Myrtales pollen with Light, Scanning Electron and Transmission Electron microscopy provides an ideal image database for this continuous morphological approach. Previous discretization of shape and ornamentation for phylogenetic analysis and character mapping [40, 53] was done for practical reasons despite known issues of categorizing continuous pollen traits (*e*.*g*., shape of the pollen as “more or less oblate” in equatorial view). Lastly, the controversial terminology surrounding pseudocolpi [62], not widespread in angiosperms but a synapomorphy for Myrtlaes [52], could benefit from additional tools that are able to quantify variation across the order.

Specifically, we ask the following questions using morphometrics of pollen in this phylogenetic framework for Myrtales: Is pollen of Myrtaceae and Onagraceae “distinctively oblate” in equatorial view as has been suggested and is this the result of convergent evolution? What important transitions in pollen shape and size have occurred during the evolutionary history of the group? Is there a signature of climatic niche space as captured through the latitudinal gradient on pollen size and shape evolution?

## Materials and methods

**Fig 1:**
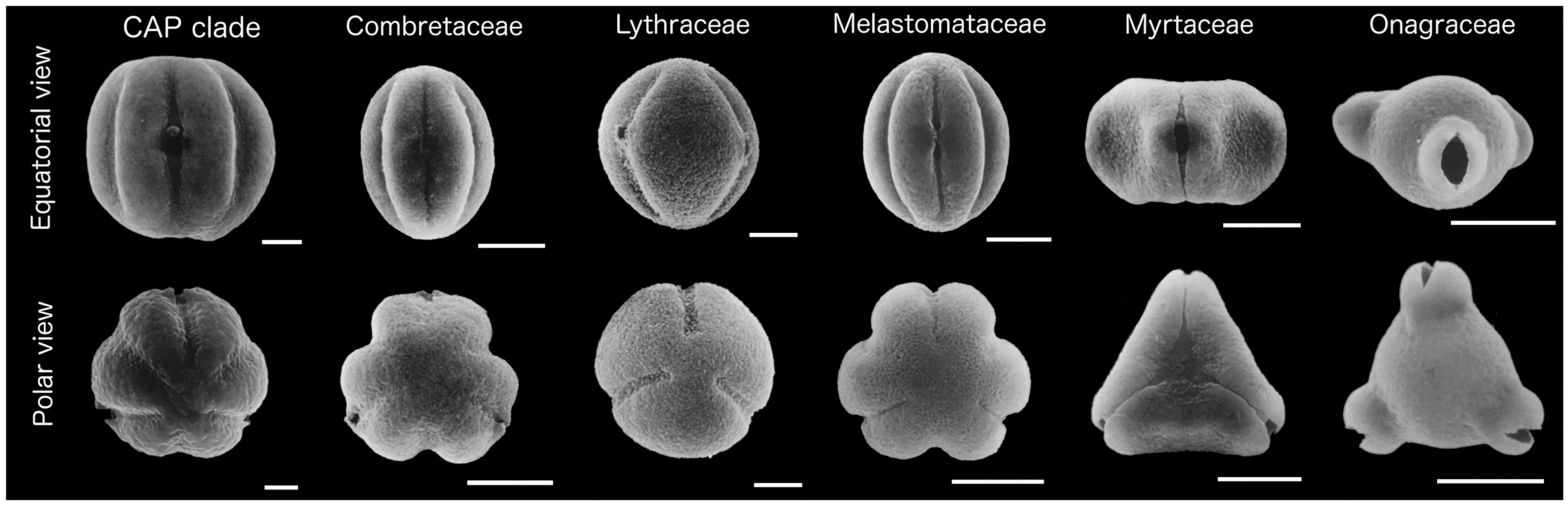
Examples of pollen grains in Myrtales. Scanning electron micrographs of pollen grains from selected species of Myrtales. Representing the CAP clade is *Saltera sarcocolla*; *Bucida macrostachya* in equatorial view and *Conocarpus erecta* in polar view (Combretaceae); *Heimia salicifolia* (Lythraceae); *Miconia alypifolia* in equatorial view and *Miconia caesia* in polar view for (Melastomaceae); *Tristania conferta* (Myrtaceae); *Calylophus toumeyi* (Onagraceae). Scale bars are 5 um except for Onagraceae which is 50 *um*. Adapted from [50] with permission from the Annals of the Missouri Botanical Garden Press.

In order to quantify shape and size of pollen in Myrtales, images of pollen grains in both equatorial and polar view (see Fig 1) were extracted for morphometric analyses and measuring from the following literature sources: [27, 50, 56, 61–104]. When the view was not clearly equatorial or polar, those grains were excluded from the analyses.

### Pollen size

Images of pollen grains from the literature were measured using the software ImageJ [105]. Four measurements were taken for each pollen grain, two for each view (Fig 1). In polar view, grains were consistently positioned with one aperture pointing upward and two apertures placed at the base. A linear measurement was taken from the aperture vertically down the middle of the two opposing apertures to represent the length in polar view. A measurement at the widest point was taken to represent width in polar view. In equatorial view, the apertures were used to guide a horizontal and linear measurement of the widest part of the grain. Prior to comparing pollen size by family within Myrtales, a Shapiro-Wilk test of normality was conducted with the stats package for R [106]. As the test showed that none of the four pollen measurements were normally distributed, they were subsequently log transformed. Pollen size was compared between groups using approximate randomization tests in the R package coin [107], with post hoc multiple comparisons implemented in the R package multcompView [108] and functions available in rcompanion [109]. Double boxplots to visualize size variation in both the equatorial and polar view of the pollen were constructed with the package boxplotdbl [110].

### Pollen shape

After extracting images of pollen grains from the literature, they were outlined using GIMP 2.8 (http://www.gimp.org). In order to quantify shape of the pollen, we conducted analysis of outlines with elliptic Fourier transformations on both the equatorial and the polar views independently with the R package Momocs [37]. To this end, the outlines are read into R and converted to lists of coordinates that describe them and then subjected to elliptic Fourier analysis (EFA) using normalization of the Fourier coefficients for rotation, translation, size and orientation. Small amounts of noise between outline halves were removed using the rm_asym and rm_sym functions in Momocs. After, a Principal Components Analysis (PCA) is conducted to summarize variation in the harmonic coefficients resulting from the EFA. A total of 32 harmonics which contributed 99% of harmonic power were used in the PCA. We opted for EFA instead of a landmark based approach because the only reliable homologous points in the pollen grains are the pores, and these result in just three landmarks in polar view and sometimes, depending on the image, one single landmark in equatorial view. For this study we were particularly interested in the area between the pores, thus the landmark based approach was insufficient to capture variation in this area. EFA on the other hand places many coordinates all around the outline and can extract very accurate information form curved areas such as in between the pollen pores. A multivariate analysis of variance (MANOVA) was done in Momocs to test for significant differences between the families using the first 15 principal components scores resulting from the EFA, which for both pollen views correspond to more than 95% of the variation. All pollen outlines and R code needed to reproduce the analyses are available at the Dryad Digital Repository (http://dx.doi.org/10.5061/dryad.j17pm).

## Comparative methods

### Phylogeny

**Fig 2:**
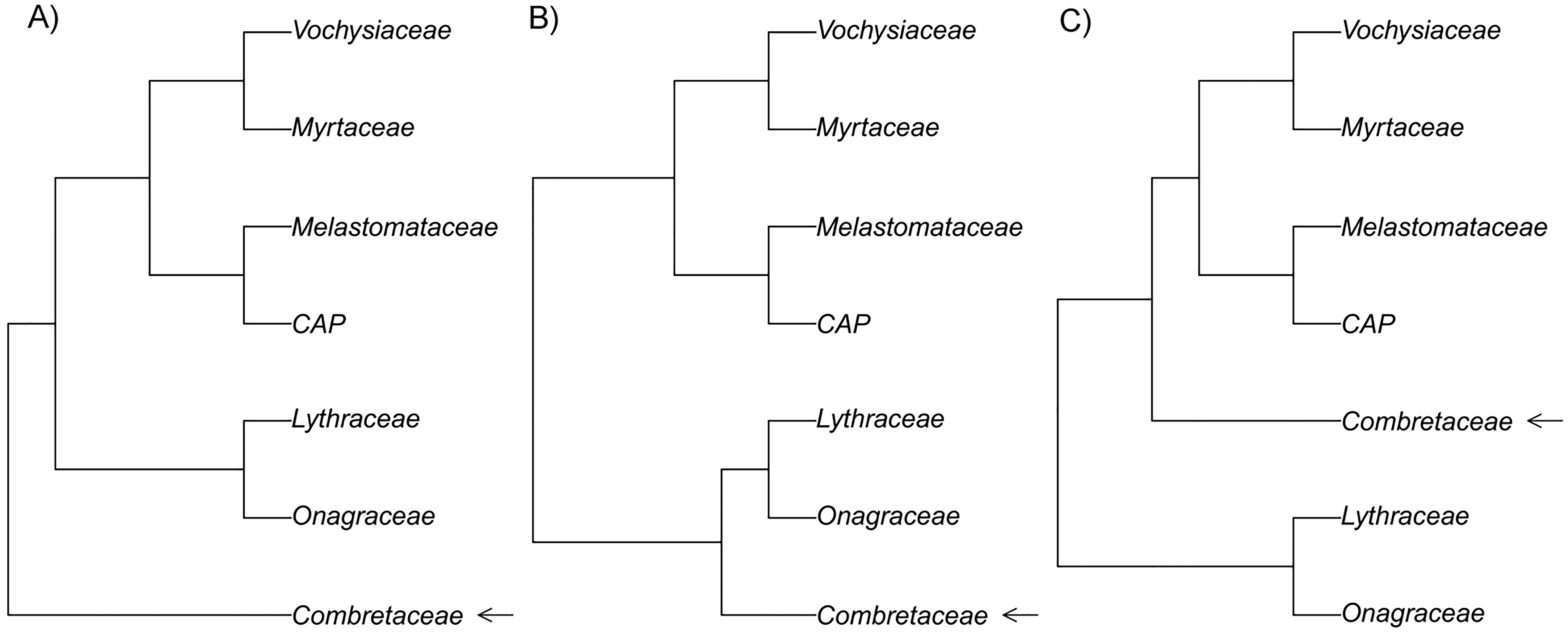
Summary of major relationships within Myrtales. Major relationships within Myrtales showing three possible placements of Combretaceae (summarized in [39]). A: Phylogeny with the family Combretaceae sister to the rest of the order. B: Phylogeny with the family Combretaceae sister to Lythraceae + Onagraceae. C: Phylogeny with the family Combretaceae as sister to rest of the order excluding Lythraceae + Onagraceae.

We generated a matrix of DNA for Myrtales from sequences available in GenBank—these included 12 regions representing 3346 tips. Among these tips we included the most likely sister to Myrtales, the Geraniales, as well as *Vitis vinifera* as the ultimate outgroup for rooting. The 10 chloroplast DNA (cpDNA) regions used were: *accD-psaI*, *atpB-rbcL*, *atpF-atpH*, *matK*, *ndhf*, *psbK-psbL*, *rbcL*, *rpl16*, *trnL-trnF*, *trnS-trnG*. We also added the nuclear ribosomal (nrDNA) spacers: external transcribed spacer (ETS) and internal transcribed spacers (ITS). DNA sequences were aligned using MAFFT version 7 [111]. For the alignment the direction of sequences were adjusted according to the first sequence and the FFT-NS-I slow, iterative refinement strategy was employed. We estimated phylogenetic trees for the cpDNA and nrDNA data independently and also as a concatenated data set. Due to the differential placement of Combretaceae seen in previous molecular phylogenetic studies (summarized in [39]), we utilized and compared pollen evolution across three different backbone topologies varying the placement of Combretaceae (Fig 2). Phylogenies were generated using Maximum Likelihood (ML) under the GTR+Γ model of sequence evolution using RAxML [112] run in the CIPRES Science Gateway v.3.3 [113]. The best ML trees from the three analyses were time-calibrated using the range of ages reported for the crown of the order Myrtales and for the crowns of each of the major clades (families) within the order [39]. The dating of the phylogenies was done using penalized likelihood as implemented in the software treePL [114]. The settings for the treePL runs included the thorough option to iterate until convergence, as well as the randomcv option to perform cross validation. In order to check the taxonomy of the tips of the phylogeny, we queried the Taxonomic Names Resolution Service (TNRS) [115] and updated any outdated names or synonyms.

### Reconstructing pollen shape and size across Myrtales

The four variables analyzed in downstream applications were pollen size as represented by log-transformed pollen length and width in polar view, PC1 of pollen shape in polar view, and PC1 of shape in equatorial view. Pollen size in equatorial view was not analyzed due to the large amount of missing data relative to that of pollen size in polar view and the similarity in the distribution of the available data to that of pollen size in polar view. Given the different numbers of species in the phylogeny that match either the shape or size data, we generated three separate datasets of different sizes for downstream analyses. The first two with all taxa that match either the shape and size data, and a third dataset in which only species with all shape and size data were retained. We used these data sets to explore the evolutionary dynamics of pollen in Myrtales as described in the “Modeling shifts” section below, and also used a phylomorphospace approach to visualize the exploration of morphospace with the phylogeny projected in said space using the library phytools for R [116].

We also tested the hypothesis of a relationship between equatorial and polar shape due to developmental constraints by using phylogenetic generalized least squares regression (PGLS) as implemented in the R package phylolm [117]. Using the same approach, we tested for allometry using pollen length in polar view and pollen shape in polar view. For both tests we compared the fit of the Brownian Motion (BM) evolutionary model and the Ornstein-Uhlenbeck (OU) process with the ancestral state estimated at the root (“OUfixedRoot”) as the phylogenetic models for the error term. PGLS models were constructed for the three phylogenies to test for sensitivity of topological uncertainty on the results.

### Modeling shifts in pollen evolution using Ornstein-Uhlenbeck process

To assess the role of selection acting on pollen traits during the evolution of Myrtales, we modeled shifts in pollen shape and size independently, as well as in a total evidence analysis with the four variables together and on the three phylogenies under an Ornstein-Uhlenbeck (OU) process. The OU process is a convenient way to model the evolution of continuous traits that allows the detection of shifts to different morphological regimes, each with different adaptive optimum, along the edges of a time calibrated phylogeny [118–122]. To graphically represent the different regimes, the edges of the phylogeny are painted with different colors corresponding to the different regimes. If no shifts are detected during the analysis and a single regime is found across the tree, then evolution would be occurring under a Brownian Motion model. We used the R package l1ou, which uses the LASSO (Least Absolute Shrinkage and Selection Operator [123]) to detect the best shift configuration [122]. An analysis with l1ou requires a phylogeny and one to many continuous traits for which we want to test for shifts. One of the advantage of this approach is that it does not require a priori identification of the location of the shifts on the phylogeny. The shift detection procedures were run on each of the three topologies. In order to avoid the over-fitting of shifts, we used the phylogenetic Bayesian Information Criterion (pBIC) to select the number of shifts. This recently developed model selection criterion accounts for the phylogenetic correlation between species and has lower false shift detection rate than criteria such as AIC [122, 124]. The maximum number of shifts to detect was set to 50 and a non-parametric bootstrap procedure was used to assess the support for the shifts that were detected in the initial search. This bootstrap procedure which is also implemented in l1ou, takes as input the best shift configuration detected by l1ou and calculates phylogenetically uncorrelated standardized residuals for each node of the phylogeny. These residuals are sampled with replacement and mapped back onto the tree creating bootstrap replicates [122]. Each bootstrap replicate was also analyzed under the pBIC model selection criterion. An additional step was run in l1ou to identify convergence to the same morphological regime. In this step similar regimes are collapsed into convergent regimes that are then painted with the same color on the edges of the tree. During this step, we also used the pBIC model selection criterion.

### Testing linkage of ecological space and pollen features

To explore the role climate might have had in pollen shape and size evolution in Myrtales (see [125]), we examined in a phylogenetic framework the correlation of latitude and pollen features. Latitudinal gradients can be considered coarse proxies for temperature and precipitation when examining plant responses to climate, and many life-history traits of plants vary along these gradients [126–128]. We characterized the distributions for species of Myrtales by mining the Global Biodiversity Information Facility (GBIF; www.gbif.org). Using conservative approaches [129], we downloaded georeferenced samples and filtered them for inaccurate and ambiguous data (*e*.*g*., duplicate records, accessions clearly outside of known species ranges, accessions with ambiguous taxonomy). The mean absolute latitude value for each species was then calculated and used in downstream analyses. We tested the hypothesis that latitude is correlated with pollen morphology in Myrtales using PGLS regression with the R package phylolm [117]. Here we also compared the fit of the Brownian Motion (BM) and Ornstein-Uhlenbeck (OU) evolutionary models. As with the other phylogenetic regressions, PGLS models were constructed for the three phylogenies to test for sensitivity of topological uncertainty.

## Results

### Pollen size

**Fig 3:**
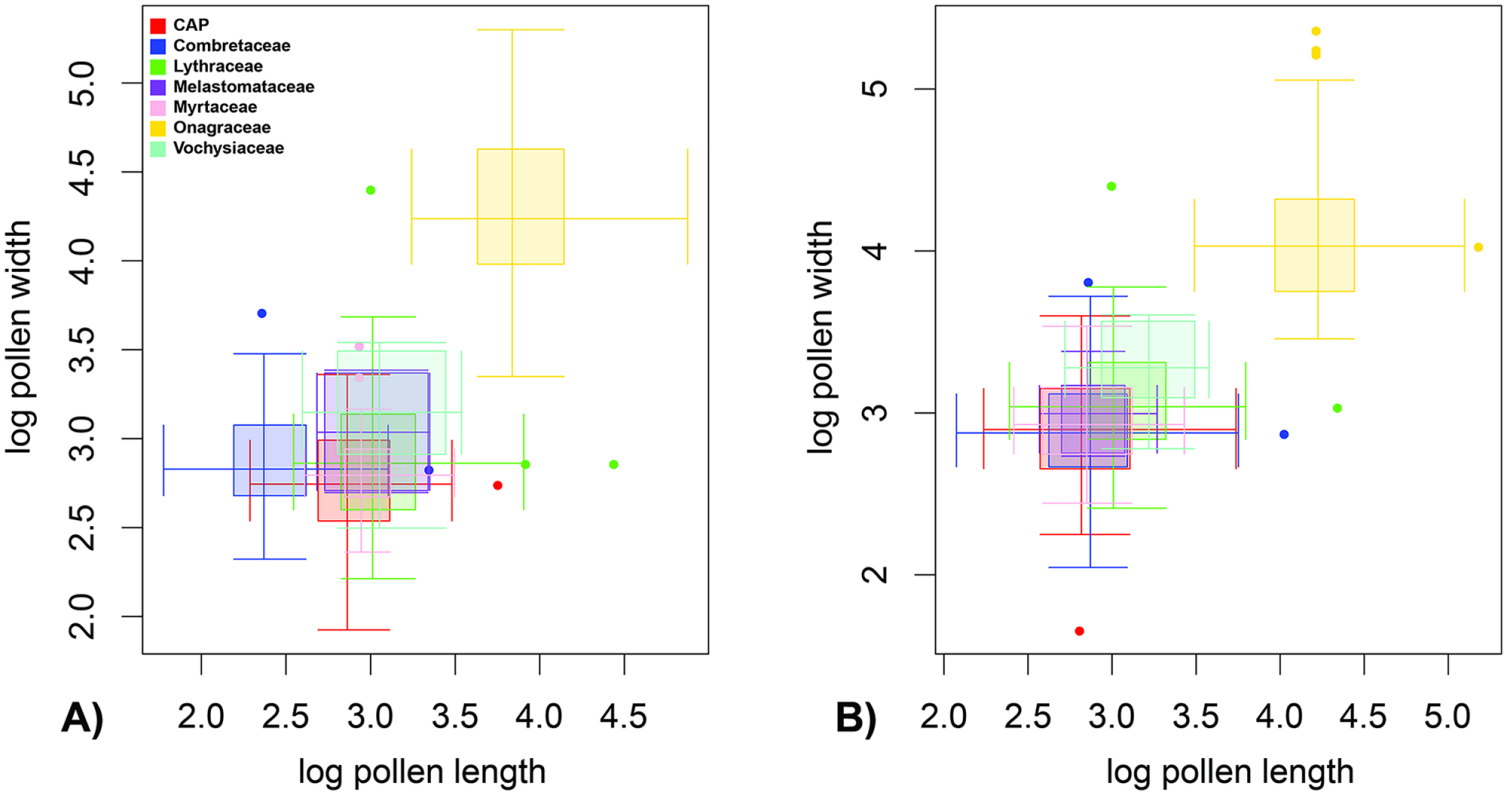
Pollen size of Myrtales. Double box-plots of pollen size of Myrtales in equatorial (A) and polar (B) views colored by family. The families Alzateaceae, Crypteroniaceae, and Penaeaceae are collectively included under the “CAP clade”.

Pollen grains from 293 and 468 species were measured for size from the literature for equatorial and polar views, respectively. Onagraceae have larger pollen grains than all other families of Myrtales (Table 1, Fig 3). Myrtaceae have smaller pollen than other families in length of pollen in equatorial view, and Melastomataceae in pollen width in equatorial view (Table 1, Fig 3). These marked differences suggest shifts in pollen size along the stem lineages of some families. Families in the CAP clade (Crypteroniaceae, Alzateaceae, Penaeaceae), as well as Combretaceae, Lythraceae, Melastomataceae and Vochysiaceae in general have relatively small pollen.

**Table 1:** Mean and standard deviation of pollen size in Myrtales (in microns). Letters in parentheses indicate significant difference between groups in post hoc multiple comparison.

	Equatorial view					Polar view				
		Pollen length		Pollen width			Pollen length		Pollen width	
Clade	n	M	SD	M	SD	n	M	SD	M	SD
**CAP**	4	21.78 (ac)	7.47	22.03 (ab)	8.13	5	18.64 (ab)	5.35	20.84 (abc)	5.89
**Combretaceae**	25	20.29 (ac)	4.94	17.53 (ab)	5.27	24	18.21 (a)	5.36	20.05 (ab)	5.92
**Lythraceae**	68	23.78 (c)	11.34	20.36 (b)	10.38	43	23.87 (b)	11.25	24 (bc)	11.64
**Melastomataceae**	96	19.05 (a)	5.84	16.35 (a)	4.93	90	18.28 (a)	6.72	19.37 (a)	6.6
**Myrtaceae**	66	11.61 (b)	3.94	18.39 (b)	5.64	237	18.65 (a)	6.63	18.89 (a)	6.1
**Onagraceae**	28	55.25 (d)	25.58	85.19 (c)	45.95	62	74.11 (c)	30.02	68.48 (d)	40.16
**Vochysiaceae**	6	23 (ac)	8.23	24.11 (b)	8.59	7	25.75 (b)	8.49	27.86 (c)	8.4

### Pollen shape

**Fig 4:**
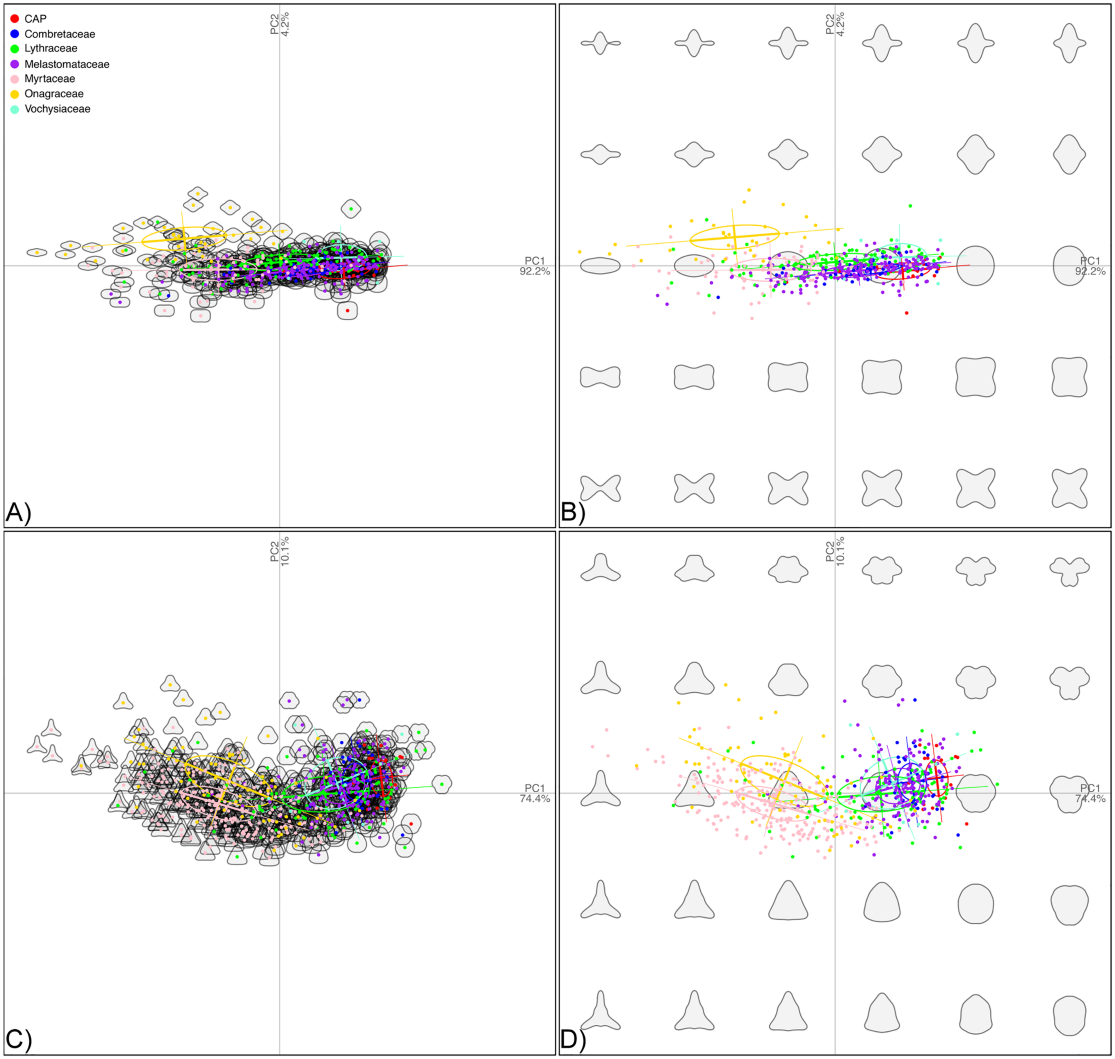
Morphospaces of pollen shape in Myrtales grouped by family. Morphospaces of pollen shape in Myrtales grouped by family. The families Alzateaceae, Crypteroniaceae, and Penaeaceae are collectively included under the “CAP clade”. A: Empirical morphospace of pollen shape variation in equatorial view. B: Theoretical morphospace of pollen shape variation in equatorial view. C: Empirical morphospace of pollen shape variation in polar view. D: Theoretical morphospace of pollen shape variation in polar view.

The elliptic Fourier analysis (eFa) of outlines in equatorial view included pollen from 444 species. 92.2% of the variation is explained in the first PC, and 4.2% in the second PC. In the first component, pollen varies in equatorial view from oblate on one extreme to rounded on the other extreme (Fig 4A and 4B). Variation in the second component describes a pronounced central body of the pollen grains with narrow ends on one extreme, to grains that are wider at the edges and narrower in the middle (Fig 4A and 4B). A multivariate analysis of variance shows significant difference between the shapes of pollen grains in equatorial view among the different families within Myrtales (*F* (6, 437) = 9.9, *p* = <0.001).

The elliptic Fourier analysis of outlines in polar view included pollen grains from 583 species. 74.4% of the variation is explained in the first PC, and 10.1% in the second PC. In the first component of polar view, pollen varies from triangular, with the area between colpi being sunken on one extreme of the variation, to more rounded pollen including a small indentation in the are between the colpi on the other (Fig 4A and 4D). Variation in the second component describes the pollen pores going from pointed on one extreme to sunken inward on the other (Fig 4). A multivariate analysis of variance shows significant difference between the shape of pollen grains in polar view among the different families within Myrtales (*F* (6, 576) = 12.8, *p* = <0.001).

### Phylogeny

The 12 region DNA matrix data set included 17507 characters representing 3346 tips and contained 77% missing data. The 10 cpDNA regions represented 2262 species and contributed 14042 characters, and the two nrDNA markers represented 2774 species and was 3465 characters in length. The phylogenies resulting from analyzing cpDNA and nrDNA independently both recover the order Myrtales as monophyletic as well as all the major families and the CAP clade. In general both topologies lack support in the backbone of the trees. The ML phylogeny resulting from the concatenated matrix recovered Myrtales and its major families as monophyletic but with low or no support for the main relationships among them. Although topology C (see Fig 2) was recovered, the differential placement of Combretaceae among the three topologies had no support. The resulting phylogenies are available from the Dryad Digital Repository (http://dx.doi.org/10.5061/dryad.j17pm).

### Reconstructing pollen shape and size across Myrtales

**Fig 5:**
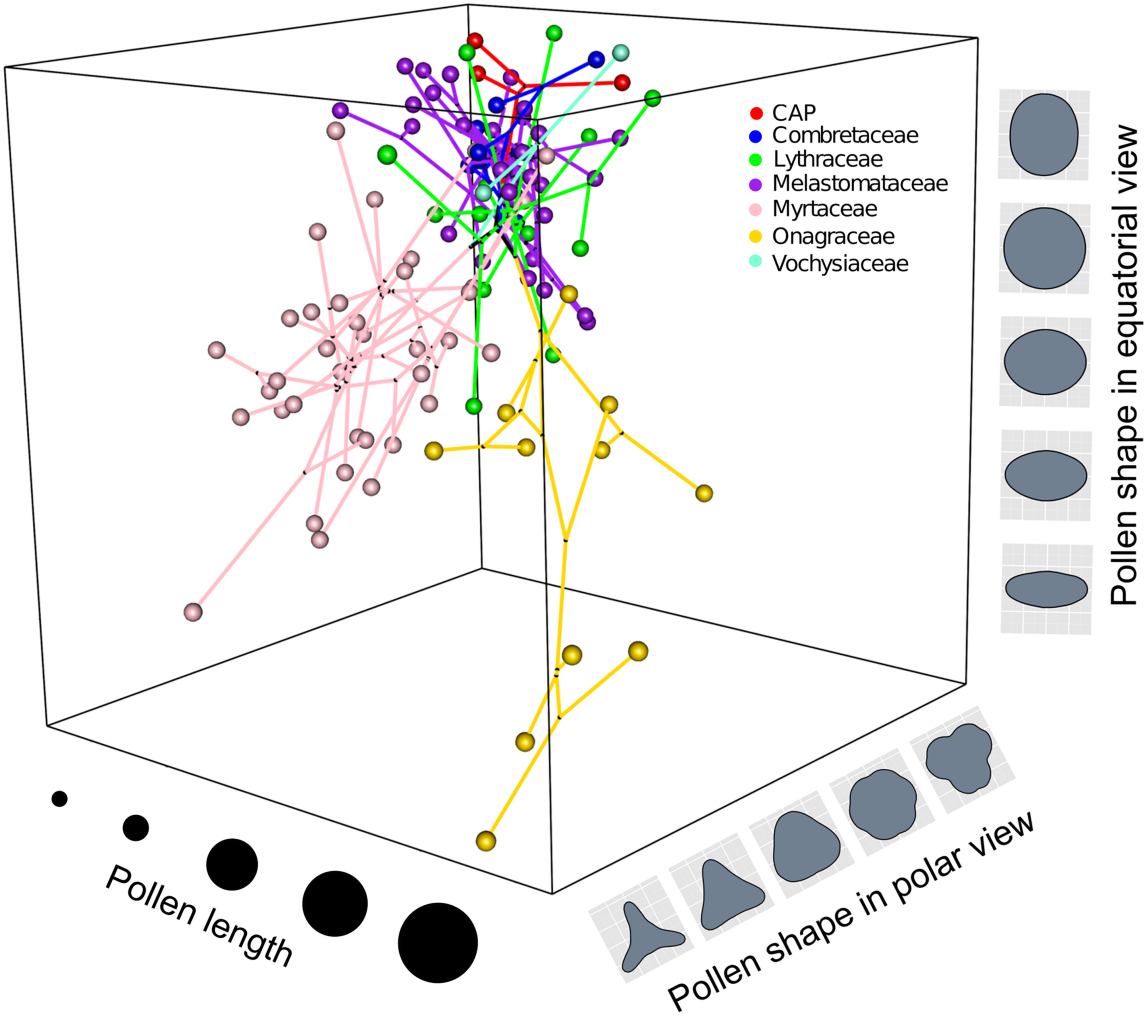
Phylomorphospace in three dimensions of Myrtales pollen traits using phylogeny A. Phylomorphospace in three dimensions including 112 species of Myrtales with pollen size represented by log transformed pollen length in polar view and pollen shape in equatorial and polar views represented by PC1 of each.

The number of species in the pollen data set that matched tips in the phylogeny was 235 for size, 173 for shape, and 112 for both shape and size. The family with the least matches was Vochysiaceae. To improve this match we used *Qualea rosea* as a phylogeny place holder for data available of *Qualea cryptantha*. A phylomorphospace in three dimensions including the two axes of shape and one of size shows the exploration of morphological space by pollen of Myrtales using phylogeny A (Fig 5). The Myrtaceae and Onagraceae individually extend into similar shape space determined by the equatorial and polar views, but they do so in quite different regions of size morphospace, with Myrtaceae and Onagraceae having the smallest and largest pollen, respectively, within Myrtales. The rest of the families are concentrated in a corner of morphospace and show little variation.

## Comparative tests

### Correlation between equatorial and polar view and allometry

For PGLS regression a comparison of both BM and OU model fits by AIC revealed the best model to be the OU in the three different trees for tests of allometry as well as developmental constraint (Table 2). Both the test of the developmental constraint hypothesis as well as the allometry hypothesis were significant across the three topologies (Table 2).

**Table 2:** Phylogenetic generalized least squares (PGLS) regression models of pollen traits. **, <0.005; ***, <0.001.

Test	Phylogeny	Model	Slope estimate	AIC	*t*	*P-value*
**Allometry**	A	BM	-0.035	-396.303	-4.252	0.001***
		OU	-0.033	-415.832	-3.939	0.001***
	B	BM	-0.045	-354.044	-4.563	0.001***
		OU	-0.038	-389.904	-4.039	0.001***
	C	BM	-0.041	-383.142	-5.578	0.001***
		OU	-0.038	-407.107	-4.873	0.001***
**Developmental constraint**	A	BM	0.02	-379.48	0.46	0.643
		OU	0.30	-409.95	9.70	0.001***
	B	BM	-0.11	-344.48	-3.18	0.002**
		OU	0.28	-401.56	8.90	0.001***
	C	BM	-0.04	-356.30	-1.02	0.311
		OU	0.30	-407.35	9.41	0.001***

### Modeling pollen evolution under an OU process

**Fig 6:**
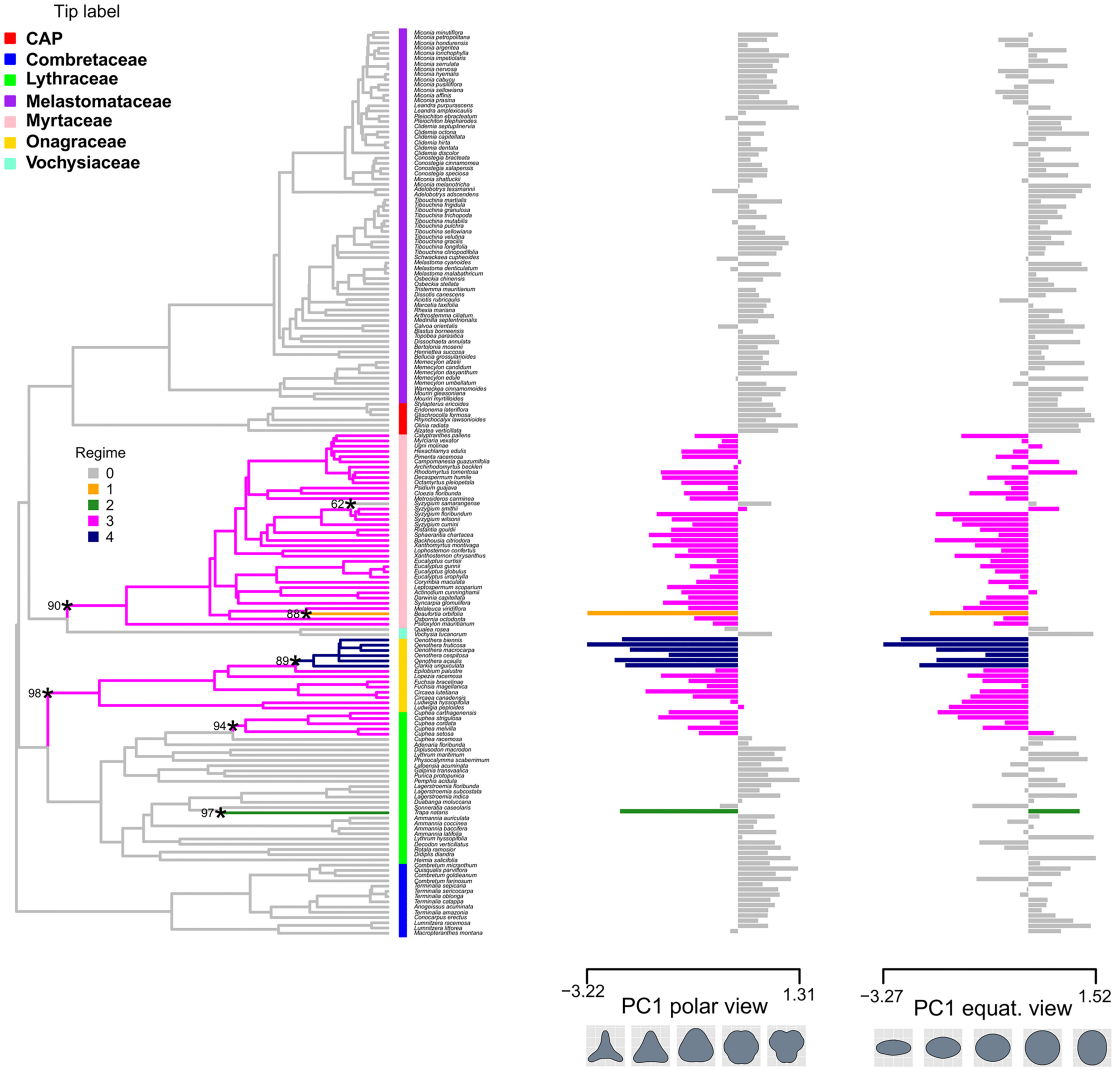
Shifts in shape during the evolutionary history of Myrtales on topology. Shifts in shape during the evolutionary history of Myrtales on topology A. The color of the edges of the tree and the bars of the bar plot indicate the regime number of that clade. Asterisks highlight edges where shifts occurred and numbers at their side indicate bootstrap support for the corresponding shift.

The maximum set number of 50 shifts was not reached in any of the three analyses (shape, size, or shape and size together) with l1ou using the three phylogenies (9 analyses total). The l1ou analysis of pollen shape detected the same 7 shifts on each of the three topologies (Fig 6, S1 and S2 Figs). Across the three trees, the background regime involves species with more rounded to pseudocolpate pollen that subsequently tends to shift to triangular pollen. Of the few shifts on edges subtending larger clades, all three analysis detected a shift to more triangular pollen in the MRCA of Myrtaceae and the MRCA of Onagraceae. The three analyses also detected convergent evolution to the same regime by Myrtaceae, Onagraceae, and *Cuphea* in the Lythraceae. Another consistent shift across trees was detected in the MRCA of the tribe Onagreae including the genera *Clarkia* and *Oenothera*, which evolved more concave areas in between the pollen pores as well as flatter equatorial shape. Singleton shifts tomore triangular pollen were detected in *Trapa natans* of the Lythraceae and *Beaufortia orbifolia* of Myrtaceae and to more rounded, elliptical pollen in *Syzygium sarangense*. Bootstrap support for shifts in shape were high with only the shift on the edge leading to *Syzygium sarangense* receiving less than 80% support (Fig 6).

**Fig 7:**
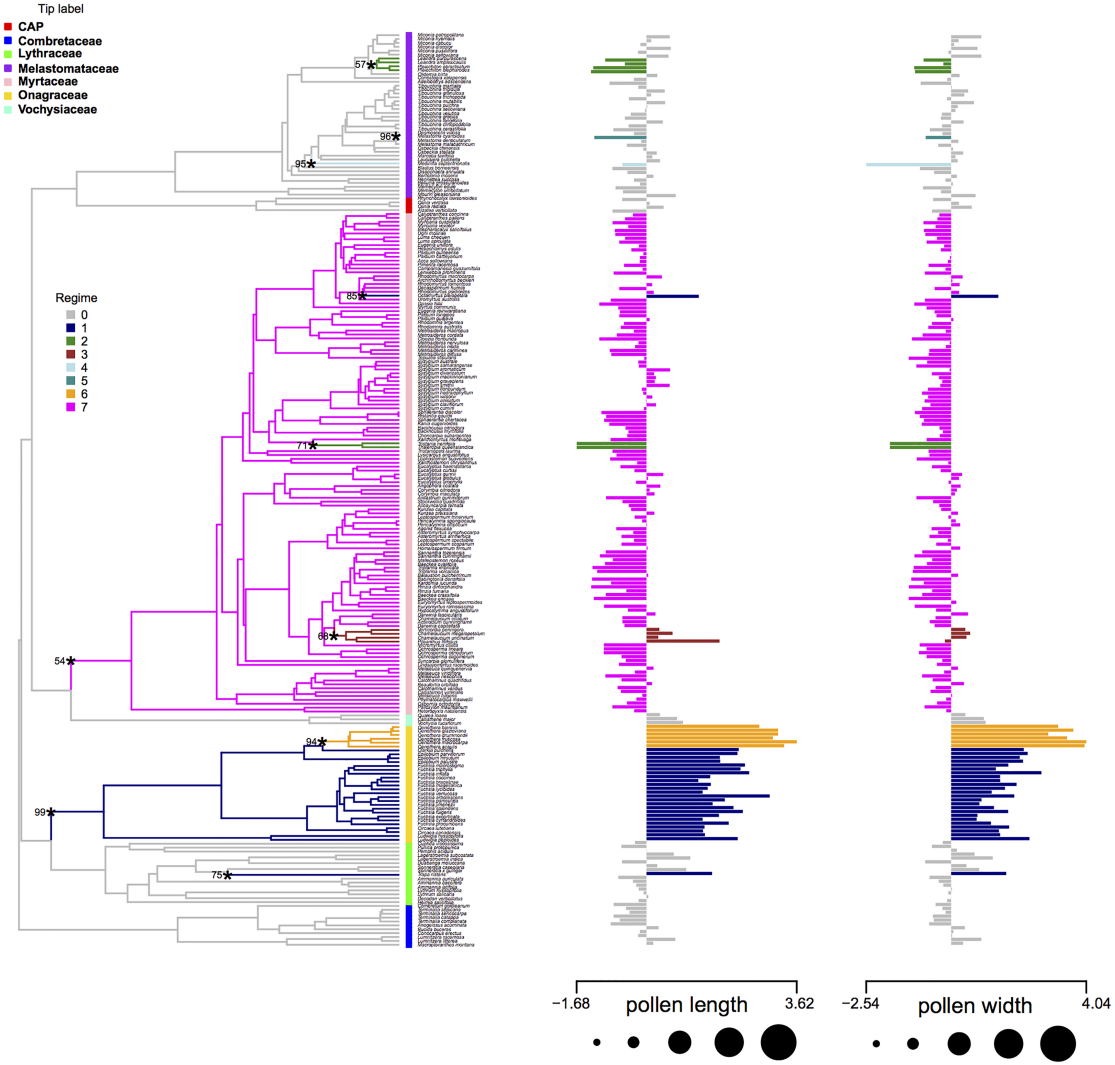
Shifts in size during the evolutionary history of Myrtales on topology. Shifts of size during the evolutionary history of Myrtales on topology A. The color of the edges of the tree and the bars of the bar plot indicate the regime number of that clade. Asterisks highlight edges where shifts occurred and numbers at their side indicate bootstrap support for the corresponding shift.

The l1ou analysis of size data detected 10 shifts on topology A, 10 on topology B, and 9 in topology C (Fig 7, S3 and S4 Figs). Across the three analyses, like in the shape data, the shifts were consistent across the three trees containing the same taxa except that topology C lacked the shift in the MRCA of the sister pair *Thaleropia queenslandica* and *Tristania neriifolia* in Myrtaceae. Most shifts were on edges leading to singleton taxa or small clades, except for a shift to smaller pollen detected in the common ancestor of Myrtaceae, and a shift to larger pollen in Onagraceae. An additional shift to even larger pollen was detected in the MRCA of the genus *Oenothera* within Onagraceae. Bootstrap support for shifts in the size only data was generally moderate to high except notably in the shift to smaller size in the MRCA of Myrtaceae. A few cases of convergence in the large size of pollen were detected, including *Trapa natans* converging to the same regime with most of Onagraceae, and in two of the topologies also with *Octamyrtus pleiopetala*.

**Fig 8:**
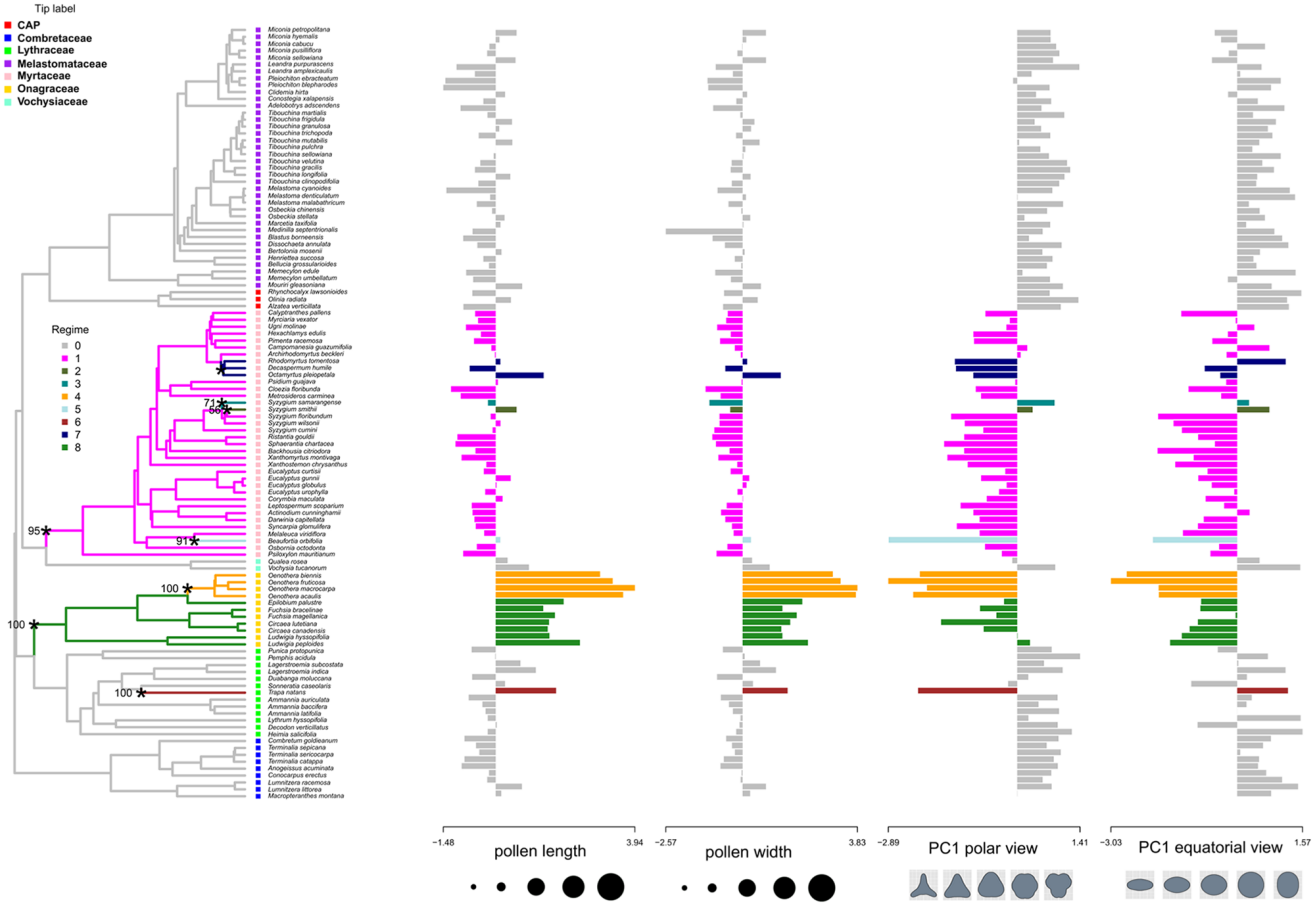
Shifts of size and shape variables in the evolutionary history of Myrtales. Shifts of size and shape variables modeled together during the evolutionary history of Myrtales on topology A. The color of the edges of the tree and the bars of the bar plot indicate the regime number of that clade. Asterisks highlight edges where shifts occurred and numbers at their side indicate bootstrap support for the corresponding shift. Only shifts with more than 50% bootstrap support are annotated. Bar plots next to the tree represent the trait values.

Finally, the l1ou analysis with shape and size data included together detected 8 shifts on topology A, 6 on topology B, and 10 in topology C (Fig 8, S5 and S6 Figs). In general the shifts are similar than in the previous analyses with changes between them mostly seen in differences in convergent regimes as well as bootstrap support. Notably on larger clades, shifts were detected in the MRCA of the Myrtaceae to smaller, more triangular and oblate pollen, and in Onagraceae to larger and more triangular and oblate pollen. In these analyses with all the data, convergence was not detected between Myrtaceae and Onagraceae do to the size difference of the pollen between the two families. One difference in the analysis on topology C was the detection of two shifts at the edges following the MRCA of the family Onagraceae instead of a the edge preceding this MRCA. The bootstrap analysis on the other hand recovered support for a shifts at this suspected, preceding edge. This result is not totally unexpected given shift detection can suffer from identifiability problems (Fig 1 of [122] illustrates this issue). Additional shifts were detected in a few singleton taxa as well as in the MRCA of the genus *Oenothera*. Bootstrap support for shifts was high except for some shifts nested deep within Myrtaceae in topologies A and C.

### Testing linkage of ecological space and pollen features

**Fig 9:**
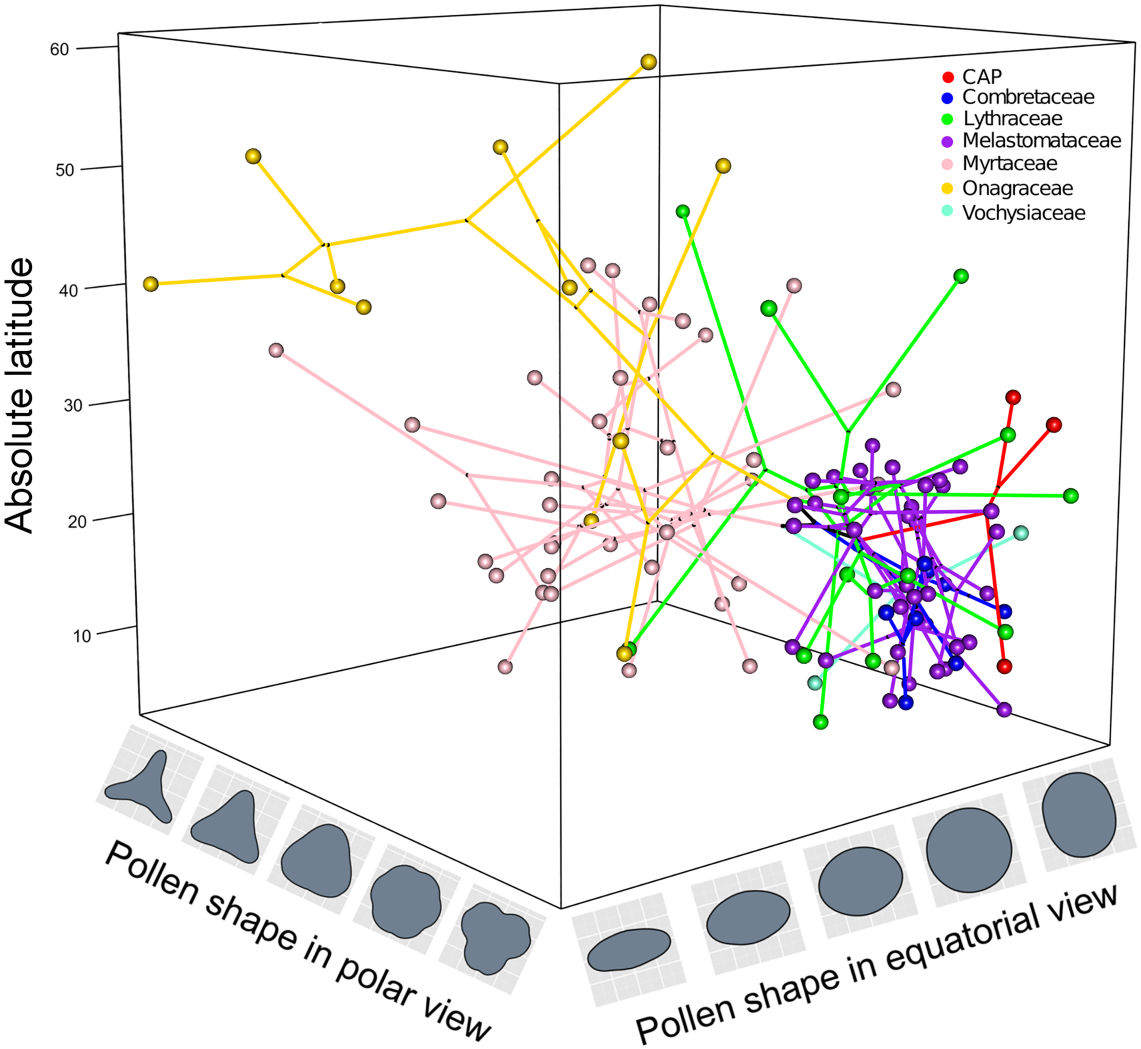
Phylomorphospace in three dimensions of Myrtales pollen shape traits and latitude. Phylomorphospace in three dimensions including 109 species of Myrtales including latitude and pollen shape in equatorial and polar views represented by PC1 of each.

A total of 1,896,305 specimens were retrieved from GBIF. This number was reduced to 813,227 after cleaning. This data set resulted in mean latitude values for a total of 10,347 species. The number of species for which there was a match between the latitudinal data, pollen data and the phylogeny was 109. The distribution of mean absolute latitude with respect to both axes of shape with the phylogeny projected inside shows the exploration of morphospace mainly by the Myrtaceae and Onagraceae (Fig 9). Phylogenetic least squares regression under the OU model was the best fitting model across the three phylogenies when comparing AIC scores. PGLS regression tests of a correlation between latitude and pollen size were not significant but those of shape were significant (Table 3).

**Table 3:** Phylogenetic generalized least squares (PGLS) regression models of pollen traits and absolute latitude. ***, <0.001.

	Phylogeny	Model	Slope estimate	AIC	*t*	*P-value*
**Shape**	A	BM	-0.002	73.924	-0.851	0.396
		OU	-0.005	-153.009	-4.821	0.001***
	B	BM	-0.001	66.237	-1.010	0.314
		OU	-0.005	-155.797	-4.665	0.001***
	C	BM	-0.001	37.990	-0.933	0.352
		OU	-0.005	-163.062	-4.867	0.001***
**Size**	A	BM	0.004	475.141	0.819	0.414
		OU	0.006	450.727	1.247	0.214
	B	BM	0.002	489.263	0.411	0.681
		OU	0.005	459.261	1.049	0.295
	C	BM	0.006	519.770	1.196	0.233
		OU	0.008	482.486	1.701	0.09

## Discussion

We present here for the first time a study of pollen evolution across a broad group of seed plants, the order Myrtales, using morphometric size measurements and elliptic Fourier analysis to quantify shape variation from two-dimensional images of equatorial and polar views of the pollen. We then couple these morphometric and morphospace approaches with a phylogenetic framework of Myrtales that is time-calibrated and assess pollen evolution across and within the nine families. We utilize an Ornstein-Uhlenbeck process and test for shifts in pollen shape and size evolution and examine convergent pollen evolution within Myrtales. And finally, we examine the specific hypothesis that the latitudinal gradient which includes changes in moisture and temperature going from tropical to temperate latitudes, may be important in the evolution of pollen size and shape because of harmomegathy.

Two important aspects of this study that permitted this broad scale analysis of pollen features should be highlighted. First, we used an indispensable data set of pollen images generated by an array of palynologists and systematists [27, 50, 56, 58, 75, 100–102]. As the field of phylogenetics enters a new era of morphometric and morphospace analyses [15], the value of detailed data and images from monographic works will be immense. Second, the development of a large molecular phylogeny of the Myrtales was critical. The most extensive phylogenetic sampling to date of the order in terms of species and gene regions was presented in [39], which included 102 species and six gene regions across three genomes with 98% cell coverage. In order to match tips on a Myrtales phylogeny to species with existing pollen images, a phylogeny with over 3300 tips from 12 gene regions was necessary but with resulting 77% missing cell coverage. However, the relationships between and within families recovered in our large phylogeny were essentially congruent with that from [39] except for the (weak) placement of Combretaceae. Our subsequent down-stream analyses thus used three different phylogenetic frameworks differing in the position of this family. The following discussion is largely based on the phylogeny (topology A) placing Combretaceae sister to the rest of Myrtales given that results across the three phylogenies were similar [39].

### Evolution of pollen size in Myrtales

The results on pollen measurements highlight that most families in Myrtales (including the Myrtales crown) have small pollen (Figs 3 and 5), a conclusion that is not surprising given that pollen is under strong selective pressure to be small [130]. However, the size data analysis under the OU process revealed that a shift in pollen size occurred in the stem of Onagraceae, which have larger pollen grains than other families within the order (Fig 7), a result hinted at [50]. In fact, one species of this genus, *Oenothera biennis*, was found to have the largest pollen among extant plants in a recent survey of all pollen producing plants [130].

Several hypotheses have been put forth to explain evolutionary changes in pollen size. First, it has been suggested that polyploidy and/or genome size is a predictor of pollen size and they are linked to larger pollen [130, 131]. A recent study, however, found no relationship between pollen size and genome size when controlling for phylogenetic history of 464 species spread across seed plants [130]. The data available on genome sizes for species in Myrtales (available at the Kew Plant C-Value Database; [132]) are limited. Combretaceae has the greatest variation in C-value in this data set. Thus, more focused studies on Combretaceae, as well as on Melastomataceae, with small pollen but many instances of polyploidy [43], and on Onagraceae (especially Onagreae), with large pollen and a fairly representative data set of C values [133–135], may provide better tests of a correlation of pollen size and genome size.

A second hypothesis proposes that pollen grain size is the result of biotic and abiotic pollinator preference and fluid dynamics [136]. Pollen grain size may be influenced by pollinator preference in the broad sense (*i*.*e*., wind versus animal pollination). Support for this hypothesis, for example, is found in the pollen of the clusioid clade of Malpighiales [25]. Within this order, great variability in pollen size and other characters in Clusiaceae appear correlated to high levels of diversity in floral morphology, pollination mechanisms, and pollinators. In contrast, pollen size in related Podostemoideae is relatively uniform consistent with them being water pollinated, aquatic plants. At a finer scale, no relationship between pollinator type (bees versus birds) and pollen size was found across angiosperms [137]. Interspecific pollen size variation more probably reflects differences in conditions for pollen germination, pollen-tube growth and ovule fertilization [137]. In the case of Myrtales, only a few comparisons have been made among closely related species. Pollen size differences between the closely related genera *Duabanga* and *Sonneratia* have been suggested not to be the result of pollinator mediated selection, since both are bat pollinated [138]. Taken together with the results obtained here of few shifts in pollen size during the evolutionary history of Myrtales, it seems likely that pollen size evolution in Myrtales might best be explained by phylogenetic constraints or by factors that are themselves phylogenetically constrained, but not by pollinator selection. More studies including sister species with different pollinators are needed to further understand the possible role of pollinators in the evolution of pollen size at these finer evolutionary scales.

A third hypothesis, related to the former, is that pollen size is correlated with pistil size and three reproductive processes that are related to the evolution of pollen size can be distinguished [31]. These include: (1) resource allocation to male function (trade-off between pollen grain size and number), (2) pollination (pollen removal, transport and deposition, pollinator type, etc.), and (3) post pollination processes (pollen germination and tube growth, fertilization, and pistil characteristics). In the future, careful selection of taxa throughout the Myrtales would permit testing of Torres’s hypothesis. At a first glance, however, it appears unlikely pollen size is related to style length in Myrtales because most clades have small pollen yet style length can vary significantly within clade.

### Evolution of pollen shape in Myrtales

The continuous morphological approach to reconstruct pollen grain shape in Myrtales using the Ornstein-Uhlenbeck process with l1ou [122] recovered as a background regime the pseudocolpate and prolate pollen grains common in Combretaceae, Melastomataceae and the CAP clade with subsequent shifts to more triangular and oblate grains in Myrtaceae and Onagraceae (Fig 6). This approach elucidated convergent evolution in pollen shape between the Myrtaceae and Onagraceae, a similarity previously noted [50]. This result provides evidence for the hypothesis that Onagraceae and Myrtaceae have distinctly “more or less oblate” pollen shape in equatorial view as previously suggested [53] and later mapped onto the phylogeny of the order as a discrete character [55]. Here we present the first quantitative test of the latter hypothesis. Perhaps not surprisingly, the results support correlative change between the shapes of pollen grains in equatorial and polar view throughout the evolution of Myrtales and suggest that forces of developmental constraint are present during pollen development. Importantly, although Myrtaceae and Onagraceae have independently achieved their unique and similar pollen shape, the 3-D phylomorphospace projection (Fig 5) clearly indicates they occupy distinct regions of the morphospace when pollen size is also factored in. Also, as seen in Fig 8, there is no convergence detected when pollen size is analyzed simultaneously with pollen shape with l1ou.

### Shifts in pollen shape and size in Myrtales using OU process

The results of the l1ou analyses also shed light on the shape and size of the common ancestor of the group. In all three tested Myrtales topologies, shifts are observed from the small heterocolpate grains present in Combretaceae, the CAP clade and Melastomataceae to more triangular grains in the Myrtaceae, Onagraceae and some Lythraceae. This result is in accord with the attribution of great importance to the presence of pseudocolpi, suggesting they had phylogenetic significance [52]. Within Onagraceae, an additional shift is consistently detected in the MRCA of the sampled of the tribe Onagreae to even larger grains with the area between the pores more concave. Lastly one of the most consistent shifts in a singleton taxon corresponded to that detected in *Trapa natans*. This shift was detected to be convergent with that at the edge leading to the MRCA of Onagraceae in the size only analysis, but not it the all data one. This convergence between the pollen of the genus *Trapa* and the Onagraceae was previously noted [50], in particular to the genus *Ludwigia*.

The detection of shifts in the evolution of pollen shape and size in the common ancestor of both Onagraceae and Myrtaceae with no to few subsequent shifts within and outside these families suggests these traits are conserved in the two families as they are in most other Myrtales. Based on the chronogram presented here [39], the pollen shifts in these two families occurred about the same time, ca. 85 Mya. The evolutionary origin of two characters previously hypothesized as significant during the diversification of the Myrtales, namely the appearance of syncolporate pollen in Myrtaceae and viscin threads in Onagraceae, may thus represent independent evidence of an important past event towards the close of the Cretaceous that simultaneously affected pollen diversification at the crown radiation of both families. The subsequent convergent shift in Onagraceae and Myrtaceae may thus be the result of contingent evolution on the origin of earlier pollen traits such as viscin threads in the Onagraceae and syncolpae in the Myrtaceae.

### Harmomegathy: Does Myrtales pollen evolution track latitude?

The last and perhaps the strongest hypothesis to explain the “form, composition, organization and architecture” of pollen grains argues that these changes are mostly a result of dealing with harmomegathic stress [32, 138, 139]. Harmomegathy, a term first used by Wodehouse [32] to indicate changes in pollen volume accommodation in response to water loss, is considered essential for life on land [33]. Pollen grains are usually exposed to dry environmental conditions once they are released from the pollen sacs, and thus have evolved the ability to fold themselves in ways that avoid dehydration [32, 33, 138, 140, 141]. Harmomegathy has only been examined in a few species within a genus or family [142, 143], including some species of Lythraceae [144]. Our study constitutes the first to evaluate the evolution of harmomegathy at the ordinal level, and in a phylogenetic context. Transition from the prolate, pseudocolpate type to the oblate, triangular types in Myrtaceae and Onagraceae appears to involve a shift from pollen that folds at the pseudocolpae when dehydrated in the case of most prolate grains, to a loss of this ability in Myrtaceae and Onagraceae which lack pseudocolpae. Importantly, the latitudinal distribution of the predominantly temperate families Myrtaceae and Onagraceae indicate that shifts in pollen shape are correlated to latitudinal shifts in both families (Fig 9). Additionally, these events are also linked to inferred long distance dispersal events between continents and exposure to a wider range of more xeric conditions during the crown radiations of both families [39]. It remains to be documented in more taxa and experimentally tested if the convergent shapes observed in Myrtaceae and Onagraceae have evolved to resolve harmomegathic stress by folding in similar ways.

### Future applications of pollen morphometric analyses

We have demonstrated the utility of morphometric approaches in clarifying the evolution of both pollen size and shape across the 116 Mya history of Myrtales. This history involved both conservatism but also remarkable evolutionary convergence in pollen features as the order diversified following shifts in both biogeography and climate. Whether pollen can be evaluated in a similar fashion in all seed plant clades and at all taxonomic scales remains to be seen. We note, however, that issues of homology may be problematic if pollen analysis is extended out from Myrtales to encompass other rosid orders. For example, Geraniales is likely the closest sister order to Myrtales [39, 145, 146], but Geraniales pollen is so distinctive [147] compared to that of Myrtales that they will be difficult to compare across so distantly related species. Perhaps not coincidentally, Geraniales, like Myrtales, have undergone a remarkable radiation in response to both climate and pollinators on an inter-continental scale [148].

Finally, we see an important role of these morphometric tools in the identification and phylogenetic placement of fossil pollen within extant clades. As microfossils generally are seen in greater abundance and earlier than macrofossils within plant fossil records, fossil pollen can be important for calibrating DNA phylogenies [102]. Like other fossils, both the taxonomic identity and the placement of fossil pollen onto branches or nodes of the tree can be contentious [146, 149–153]. The objective fashion in which elliptic Fourier analysis treats shape variables lends itself to perhaps more objective handling of fossil pollen for tree calibration. The phylogenetic framework provided here for Myrtales should allow more rigorous testing of the placements of fossil pollen recently used within the order and the family Myrtaceae [27, 39, 56, 154]. Indeed, these methods may soon more elegantly narrow down the taxonomic identity and placement of important, unknown pollen fossils, such as those found in the stomach of the Eocene (47 Mya) *Pumiliornis*, the earliest known flower visiting bird [155].

## Supporting information

S1 Fig Shifts in pollen shape variables during the evolutionary history of Myrtales on topology B. The color of the edges of the tree and the bars of the bar plot indicate the regime number of that clade. Asterisks highlight edges where shifts occurred and numbers at their side indicate bootstrap support for the corresponding shift. Only shifts with more than 50% bootstrap support are annotated. Bar plots next to the tree represent the trait values.
(TIF)

S2 Fig Shifts in pollen shape variables during the evolutionary history of Myrtales on topology C. The color of the edges of the tree and the bars of the bar plot indicate the regime number of that clade. Asterisks highlight edges where shifts occurred and numbers at their side indicate bootstrap support for the corresponding shift. Only shifts with more than 50% bootstrap support are annotated. Bar plots next to the tree represent the trait values.
(TIF)

S3 Fig Shifts in pollen size during the evolutionary history of Myrtales on topology B. The color of the edges of the tree and the bars of the bar plot indicate the regime number of that clade. Asterisks highlight edges where shifts occurred and numbers at their side indicate bootstrap support for the corresponding shift. Only shifts with more than 50% bootstrap support are annotated. Bar plots next to the tree represent the trait values.
(TIF)

S4 Fig Shifts in pollen size during the evolutionary history of Myrtales on topology C. The color of the edges of the tree and the bars of the bar plot indicate the regime number of that clade. Asterisks highlight edges where shifts occurred and numbers at their side indicate bootstrap support for the corresponding shift. Only shifts with more than 50% bootstrap support are annotated. Bar plots next to the tree represent the trait values.
(TIF)

S5 Fig Shifts of size variables and shape variables modeled together during the evolutionary history of Myrtales on topology B. The color of the edges of the tree and the bars of the bar plot indicate the regime number of that clade. Asterisks highlight edges where shifts occurred and numbers at their side indicate bootstrap support for the corresponding shift. Only shifts with more than 50% bootstrap support are annotated. Bar plots next to the tree represent the trait values.
(TIF)

S6 Fig Shifts of size variables and shape variables modeled together during the evolutionary history of Myrtales on topology C. The color of the edges of the tree and the bars of the bar plot indicate the regime number of that clade. Asterisks highlight edges where shifts occurred and numbers at their side indicate bootstrap support for the corresponding shift. Only shifts with more than 50% bootstrap support are annotated. Bar plots next to the tree represent the trait values.
(TIF)

## References

[1] Crisp MD, Arroyo MT, Cook LG, Gandolfo MA, Jordan GJ, McGlone MS, et al Phylogenetic biome conservatism on a global scale. Nature. 2009;458(7239):754–756. doi: 10.1038/nature07764 19219025 10.1038/nature07764

[2] Rabosky DL, Santini F, Eastman J, Smith SA, Sidlauskas B, Chang J, et al Rates of speciation and morphological evolution are correlated across the largest vertebrate radiation. Nature Communications. 2013;4:55–78. doi: 10.1038/ncomms2958 10.1038/ncomms2958 23739623

[3] Givnish TJ, Barfuss MHJ, Ee BV, Riina R, Schulte K, Horres R, et al Adaptive radiation, correlated and contingent evolution, and net species diversification in Bromeliaceae. Molecular Phylogenetics and Evolution. 2014;71:55–78. doi: 10.1016/j.ympev.2013.10.010 24513576 10.1016/j.ympev.2013.10.010

[4] Linder PH, Rabosky DL, Antonelli A, Wüest RO, Ohlemüller R. Disentangling the influence of climatic and geological changes on species radiations. Journal of Biogeography. 2014;41(7):1313–1325. doi: 10.1111/jbi.12312

[5] Rose JP, Kriebel R, Sytsma KJ. Shape analysis of moss (Bryophyta) sporophytes: Insights into land plant evolution. American Journal of Botany. 2016; doi: 10.3732/ajb.1500394 26944353 10.3732/ajb.1500394

[6] Thiele K. The holy grail of the perfect character: the cladistic treatment of morphometric data. Cladistics. 1993;9(3):275–304. doi: 10.1111/j.1096-0031.1993.tb00226.x 10.1111/j.1096-0031.1993.tb00226.x 34929957

[7] Wiens JJ. Character analysis in morphological phylogenetics: problems and solutions. Systematic Biology. 2001;50(5):689–699. doi: 10.1080/106351501753328811 12116939 10.1080/106351501753328811

[8] Lavin SR, Karasov WH, Ives AR, Middleton KM, Garland T Jr. Morphometrics of the avian small intestine compared with that of nonflying mammals: a phylogenetic approach. Physiological and Biochemical Zoology. 2008;81(5):526–550. doi: 10.1086/590395 18754728 10.1086/590395

[9] Klingenberg CP. Evolution and development of shape: integrating quantitative approaches. Nature Reviews Genetics. 2010;11(9):623–635. 20697423 10.1038/nrg2829

[10] Klingenberg CP, Duttke S, Whelan S, Kim M. Developmental plasticity, morphological variation and evolvability: a multilevel analysis of morphometric integration in the shape of compound leaves. Journal of Evolutionary Biology. 2012;25(1):115–129. doi: 10.1111/j.1420-9101.2011.02410.x 22070353 10.1111/j.1420-9101.2011.02410.x

[11] Viscosi V, Cardini A. Leaf morphology, taxonomy and geometric morphometrics: a simplified protocol for beginners. PLoS ONE. 2011;6(10):e25630 doi: 10.1371/journal.pone.0025630 21991324 10.1371/journal.pone.0025630 PMC3184990

[12] Beaulieu JM, Donoghue MJ. Fruit evolution and diversification in Campanulid Angiosperms. Evolution. 2013;67(11):3132–3144. doi: 10.1111/evo.12180 24151998 10.1111/evo.12180

[13] Siver PA, Wolfe AP, Rohlf FJ, Shin W, Jo BY. Combining geometric morphometrics, molecular phylogeny, and micropaleontology to assess evolutionary patterns in *Mallomonas* (Synurophyceae: Heterokontophyta). Geobiology. 2013;11(2):127–138. doi: 10.1111/gbi.12023 23331313 10.1111/gbi.12023

[14] Smith UE, Hendricks JR. Geometric morphometric character suites as phylogenetic data: extracting phylogenetic signal from gastropod shells. Systematic Biology. 2013;62(3):366–385. doi: 10.1093/sysbio/syt002 23325808 10.1093/sysbio/syt002

[15] Chartier M, Jabbour F, Gerber S, Mitteroecker P, Sauquet H, von Balthazar M, et al The floral morphospace—a modern comparative approach to study angiosperm evolution. New Phytologist. 2014;204(4):841–853. doi: 10.1111/nph.12969 25539005 10.1111/nph.12969 PMC5526441

[16] Eberle J, Myburgh R, Ahrens D. The evolution of morphospace in phytophagous Scarab chafers: no competition—no divergence? PLoS ONE. 2014;9(5):e98536 doi: 10.1371/journal.pone.0098536 24875856 10.1371/journal.pone.0098536 PMC4038600

[17] Robbins RR, Dickinson DB, Rhodes AM. Morphometric analysis of pollen from four species of *Ambrosia* (Compositae). American Journal of Botany. 1979;66(5):538–545. doi: 10.2307/2442503

[18] Lindbladh M, O’Connor R, Jacobson GL. Morphometric analysis of pollen grains for paleoecological studies: classification of *Picea* from eastern North America. American Journal of Botany. 2002;89(9):1459–1467. doi: 10.3732/ajb.89.9.1459 21665747 10.3732/ajb.89.9.1459

[19] Wrońska-Pilarek D, Boratyńska K. Pollen morphology of *Rosa gallica* L. (Rosaceae) from Southern Poland. Acta Societatis Botanicorum Poloniae. 2005;74(4):297–304. doi: 10.5586/asbp.2005.038

[20] Ahmad-Khanbeygi Z, Sheidai M, Attar F. Morphometry and palynological study of the genus *Cousinia* sect. *Cousinia* (Asteraceae) in Iran. Iranian Journal of Botany. 2011;17(2):158–166.

[21] Wrońska-Pilarek D, Jagodziński AM. Systematic importance of pollen morphological features of selected species from the genus *Rosa* (Rosaceae). Plant Systematics and Evolution. 2011;295(1–4):55–72. doi: 10.1007/s00606-011-0462-y

[22] Sauquet H, Le Thomas A. Pollen diversity and evolution in Myristicaceae (Magnoliales). International Journal of Plant Sciences. 2003;164(4):613–628. doi: 10.1086/375424

[23] Schols P, Wilkin P, Furness CA, Huysmans S, Smets E, Meerow AW. Pollen evolution in yams (*Dioscorea*: Dioscoreaceae). Systematic Botany. 2005;30(4):750–758. doi: 10.1600/036364405775097743

[24] Furness C, Banks H. Pollen evolution in the early divergent Monocot order Alismatales. International Journal of Plant Sciences. 2010;171(7):713–739. doi: 10.1086/654848

[25] Furness CA. Pollen evolution in the Clusioid clade (Malpighiales). International Journal of Plant Sciences. 2012;173(9):1055–1082. doi: 10.1086/667614

[26] Welsh M, Stefanović S, Costea M. Pollen evolution and its taxonomic significance in *Cuscuta* (dodders, Convolvulaceae). Plant Systematics and Evolution. 2010;285(1–2):83–101. doi: 10.1007/s00606-009-0259-4

[27] Thornhill AH, Crisp MD. Phylogenetic assessment of pollen characters in Myrtaceae. Australian Systematic Botany. 2012;25(3):171–187. doi: 10.1071/SB11019

[28] Leslie AB, Beaulieu JM, Crane PR, Knopf P, Donoghue MJ. Integration and macroevolutionary patterns in the pollination biology of conifers. Evolution. 2015;69(6):1573–1583. doi: 10.1111/evo.12670 25903435 10.1111/evo.12670

[29] Schols P, D’hondt C, Geuten K, Merckx V, Janssens S, Smets E. Morphocode: coding quantitative data for phylogenetic analysis. PhyloInformatics. 2004;4:1–4.

[30] Muller J. Review of shape and symmetry terminology for pollen and spores. Fourth International Palynological Conference, Lucknow (1976–77). 1978;1:194–198.

[31] Torres C. Pollen size evolution: correlation between pollen volume and pistil length in Asteraceae. Sexual Plant Reproduction. 2000;12(6):365–370. doi: 10.1007/s004970000030

[32] Wodehouse RP. Pollen grains. McGraw-Hill, New York; 1935.

[33] Blackmore S, Barnes SH. Harmomegathic mechanisms in pollen grains In: Blackmore S, Ferguson IK, editors. Pollen and spores: Form and function. London, UK: Academic Press; 1984 p. 137–149.

[34] Chong SML, Constantino-Santos DMA, Cao EP. Pollen morphometrics of four coffee (*Coffea* sp.) varieties grown in the Philippines. Philippine Journal of Crop Science. 2014;39(3):1–7.

[35] Kuhl FP, Giardina CR. Elliptic Fourier features of a closed contour. Computer Graphics and Image Processing. 1982;18(3):236–258. doi: 10.1016/0146-664X(82)90034-X

[36] Kincaid D, Schneider RB. Quantification of leaf shape with a microcomputer and Fourier transform. Canadian Journal of Botany. 1983;61:2333–2342. doi: 10.1139/b83-256

[37] Bonhomme V, Picq S, Gaucherel C, Claude J. Momocs: outline analysis using R. Journal of Statistical Software. 2014;56(1):1–24.

[38] Bonhomme V, Prasad S, Gaucherel C. Intraspecific variability of pollen morphology as revealed by elliptic Fourier analysis. Plant Systematics and Evolution. 2013;299(5):811–816. doi: 10.1007/s00606-013-0762-5

[39] Berger BA, Kriebel R, Spalink D, Sytsma KJ. Divergence times, historical biogeography, and shifts in speciation rates of Myrtales. Molecular Phylogenetics and Evolution. 2016;95:116–136. doi: 10.1016/j.ympev.2015.10.001 26585030 10.1016/j.ympev.2015.10.001

[40] Conti E, Rutschmann F, Eriksson T, Sytsma KJ, Baum DA, Harrison R. Calibration of molecular clocks and the biogeographic history of Crypteroniaceae: a reply to Moyle. Evolution. 2004;58(8):1874–1876. doi: 10.1111/j.0014-3820.2004.tb00473.x 10.1111/j.0014-3820.2004.tb00472.x 15446441

[41] Sytsma KJ, Litt A, Zjhra ML, Pires JC, Nepokroeff M, Conti E, et al Clades, clocks, and continents: historical and biogeographical analysis of Myrtaceae, Vochysiaceae, and relatives in the Southern Hemisphere. International Journal of Plant Sciences. 2004;165(S4):S85–S105. doi: 10.1086/421066

[42] APGIII. An update of the Angiosperm Phylogeny Group classification for the orders and families of flowering plants: APG III. Botanical Journal of the Linnean Society. 2009;161(2):105–121. doi: 10.1111/j.1095-8339.2009.00996.x

[43] Renner SS. A survey of reproductive biology in Neotropical Melastomataceae and Memecylaceae. Annals of the Missouri Botanical Garden. 1989;76(2):496–518. doi: 10.2307/2399497

[44] Slater AT, Beardsell D. Secondary pollen presentation in the *Chamelaucium* alliance of the Myrtaceae: a compact substigmatic ring in *Chamelaucium*. Australian Journal of Botany. 1991;39(3):229–239. doi: 10.1071/BT9910229

[45] Ladd PG. Pollen presenters in the flowering plants—form and function. Botanical Journal of the Linnean Society. 1994;115(3):165–195. doi: 10.1006/bojl.1994.1040

[46] Raven PH. A survey of reproductive biology in Onagraceae. New Zealand Journal of Botany. 1979;17(4):575–593. doi: 10.1080/0028825X.1979.10432572

[47] Lumer C. Rodent pollination of *Blakea* (Melastomataceae) in a Costa Rican cloud forest. Brittonia. 1980;32(4):512–517. doi: 10.2307/2806163

[48] Beardsell D, Obrien S, Williams E, Knox R, Calder D. Reproductive biology of Australian Myrtaceae. Australian Journal of Botany. 1993;41(5):511–526. doi: 10.1071/BT9930511

[49] Gressler E, Pizo MA, Morellato LPC. Polinização e dispersão de sementes em Myrtaceae do Brasil. Brazilian Journal of Botany. 2006;29(4):509–530. doi: 10.1590/S0100-84042006000400002

[50] Patel VC, Skvarla JJ, Raven PH. Pollen characters in relation to the delimitation of Myrtales. Annals of the Missouri Botanical Garden. 1984;71(3):858–969. doi: 10.2307/2399170

[51] Tobe H, Raven PH. An embryological analysis of Myrtales: its definition and characteristics. Annals of the Missouri Botanical Garden. 1983;70(1):71–94. doi: 10.2307/2399008

[52] Dahlgren R, Thorne RF. The order Myrtales: circumscription, variation, and relationships. Annals of the Missouri Botanical Garden. 1984;71(3):633–699. doi: 10.2307/2399158

[53] Johnson LAS, Briggs BG. Myrtales and Myrtaceae-A phylogenetic analysis. Annals of the Missouri Botanical Garden. 1984;71(3):700–756. doi: 10.2307/2399159

[54] Raven PH. The order Myrtales: a symposium. Annals of the Missouri Botanical Garden. 1984;71(3):631–632.

[55] Conti E, Litt A, Wilson PG, Graham SA, Briggs BG, Johnson L, et al Interfamilial relationships in Myrtales: molecular phylogeny and patterns of morphological evolution. Systematic Botany. 1997;22:629–647. doi: 10.2307/2419432

[56] Thornhill AH, Macphail M. Fossil myrtaceous pollen as evidence for the evolutionary history of Myrtaceae: A review of fossil *Myrtaceidites* species. Review of Palaeobotany and Palynology. 2012;176–177:1–23. doi: 10.1016/j.revpalbo.2012.03.003

[57] Thornhill AH, Popple LW, Carter RJ, Ho SY, Crisp MD. Are pollen fossils useful for calibrating relaxed molecular clock dating of phylogenies? A comparative study using Myrtaceae. Molecular Phylogenetics and Evolution. 2012;63(1):15–27. doi: 10.1016/j.ympev.2011.12.003 22197806 10.1016/j.ympev.2011.12.003

[58] Skvarla JJ, Raven PH, Praglowski J. The evolution of pollen tetrads in Onagraceae. American Journal of Botany. 1975;62(1):6–35. doi: 10.2307/2442074 10.1002/j.1537-2197.1975.tb12334.x 30139106

[59] Skvarla J, Raven P, Chissoe W, Sharp M. An ultrastructural study of viscin threads in Onagraceae pollen. Pollen et Spores. 1978;20(1):5–143.

[60] Keri C, Zetter R. Notes on the exine ultrastructure of Onagraceae and Rhododendroideae (Ericaceae). Grana. 1992;31(2):119–123. doi: 10.1080/00173139209430731

[61] Van der Hammen T, Garcia de Mutis C. The Paleocene pollen flora of Colombia. Leidse Geologische Mededelingen. 1966;35:105–114.

[62] Patel V, Skvarla JJ, Raven PH. Half pseudocolpi, a unique feature of *Olinia* (Oliniaceae) pollen. American Journal of Botany. 1983;70(3):469–473. doi: 10.2307/2443255

[63] Cos Campos D. Etude des grains de pollen des Lythracees du Perou. Pollen et Spores. 1964;6:303–345.

[64] Ting W. Pollen morphology of Onagraceae. Pollen et Spores. 1966;8:9–36.

[65] Brown CA. Pollen morphology of the Onagraceae. Review of Palaeobotany and Palynology. 1967;3(1):163–180. doi: 10.1016/0034-6667(67)90050-4

[66] Barth OM, Barbosa AF. Catálogo sistemático dos pólens das plantas arbóreas do Brasil meridional: XX—Chloranthaceae e Piperaceae. Memórias do Instituto Oswaldo Cruz. 1975;73(1–2):101–108. doi: 10.1590/S0074-02761975000100007

[67] Guers J. Combretaceae, Lythraceae, Melastomataceae, Myrtaceae. Pollen et spores d’Afrique tropical Assoc Palynologues Langue Francaise, Travaux Documents Geographic Tropical. 1974;16:86–95.

[68] Muller J. Note on the pollen morphology. Blumea. 1975;22(2):275–294.

[69] Graham SA. The American species of *Nesaea* (Lythraceae) and their relationship to *Heimia* and *Decodon*. Systematic Botany. 1977;2:61–71. doi: 10.2307/2418502

[70] Graham SA. Revision of the Caribbean genus *Ginoria* (Lythraceae), including *Haitia* from Hispaniola. Annals of the Missouri Botanical Garden. 2010;97(1):34–90. doi: 10.3417/2007028

[71] Lee S. Studies on the pollen morphology in the Lythraceae. Korean Journal of Botany. 1979;22(4):115–33.

[72] Nowicke JW, Skvarla JJ, Raven PH, Berry PE. A palynological study of the genus *Fuchsia* (Onagraceae). Annals of the Missouri Botanical Garden. 1984;71:35–91. doi: 10.2307/2399056

[73] Daghlian CP, Skvarla JJ, Pocknall D, Raven PH. *Fuchsia* pollen from the early Miocene of New Zealand. American Journal of Botany. 1985;72:1039–1047. doi: 10.2307/2443446

[74] Graham A, Graham SA. Palynology and systematics of *Cuphea* (Lythraceae). I. Morphology and ultrastructure of the pollen wall. American Journal of Botany. 1968;55:1080–1088. doi: 10.2307/2440476

[75] Graham A, Nowicke J, Skvarla JJ, Graham SA, Patel V, Lee S. Palynology and systematics of the Lythraceae. I. Introduction and genera *Adenaria* through *Ginoria*. American Journal of Botany. 1985;72:1012–1031. doi: 10.2307/2443444

[76] Graham A, Nowicke JW, Skvarla JJ, Graham SA, Patel V, Lee S. Palynology and systematics of the Lythraceae. II. Genera *Haitia* through *Peplis*. American Journal of Botany. 1987;74:829–850. doi: 10.2307/2443864 10.1002/j.1537-2197.1990.tb13543.x 30139068

[77] Graham A, Graham SA, Nowicke JW, Patel V, Lee S. Palynology and systematics of the Lythraceae. III. Genera *Physocalymma* through *Woodfordia*, addenda, and conclusions. American Journal of Botany. 1990;77:159–177. doi: 10.2307/2444639 10.1002/j.1537-2197.1990.tb13543.x 30139068

[78] Renner SS. Systematic studies in the Melastomataceae: *Bellucia*, *Loreya*, and *Macairea*. Memoirs of the New York Botanical Garden. 1989;50:1–112.

[79] Roubik DW, Moreno P. Pollen and spores of Barro Colorado Island [Panama]. Monographs in Systematic Botany from the Missouri Botanical Garden. 1991;36.

[80] Almeda F. *Stanmarkia*, a new genus of Melastomataceae from the volcanic highlands of western Guatemala and adjacent Mexico. Brittonia. 1993;45(3):187–203. doi: 10.2307/2807100

[81] Gross C. The breeding system and pollinators of *Melastoma affine* (Melastomataceae); a pioneer shrub in tropical Australia. Biotropica. 1993;25:468–474. doi: 10.2307/2388870

[82] Santos F, Souza M, Makino-Watanabe H, Borges H, Goldenberg R. Palinotaxonomia de especies brasileiras do genero *Ossaea* DC. (Melastomataceae). Polibotanica. 1997;5:1–12.

[83] Hsieh TH, Yang KC. *Sonerila* Roxb. (Melastomataceae), a new generic record for the flora of Taiwan. Taiwania. 1999;44:529–532.

[84] Buchner R, Weber M. *PalDat: a palynological database*; 2000. Available from: www.paldat.org [accessed {September} 2015].

[85] Premathilake R, Nilsson S. Pollen morphology of endemic species of the Horton Plains National Park, Sri Lanka. Grana. 2001;40(6):256–279. doi: 10.1080/00173130152987508

[86] Wang YF, Chen SH. Pollen Flora of Yuenyang Lake Nature Preserve, Taiwan. Taiwania. 2001;46(2):167–191.

[87] Wang WM, Harley MM. The Miocene genus *Fupingopollenites*: comparisons with ultrastructure and pseudocolpi in modern pollen. Review of Palaeobotany and Palynology. 2004;131(1):117–145. doi: 10.1016/j.revpalbo.2004.03.005

[88] Skvarla JJ, Rowley JR, Hoch PC, Chissoe WF. Viscin threads on pollen of *Circaea x intermedia* Ehrh. (Circaeeae: Onagraceae). Taxon. 2005;54(1):121–126. doi: 10.2307/25065308

[89] Bush MB, Weng C. Introducing a new (freeware) tool for palynology. Journal of Biogeography. 2007;34(3):377–380. doi: 10.1111/j.1365-2699.2006.01645.x

[90] APSA. The Australasian Pollen and Spore Atlas V1.0. Canberra, Australia: Australian National University; 2007. Available from: http://apsa.anu.edu.au/.

[91] Cruz-Barros MAVd, Corrêa AMdS, Gasparino EC, Paes VB. Pollinic flora of Reserva do Parque Estadual das Fontes do Ipiranga (São Paulo, Brazil). Family: 90—Melastomataceae. Hoehnea. 2007;34(4):531–552. doi: 10.1590/S2236-89062007000400008

[92] Wenhong C, Yumin S, Lumen Z. Additional notes on Melastomataceae of China. Acta Phytotaxonomica Sinica. 2007;45:587–592. doi: 10.1360/aps050006

[93] Liu YS, Zetter R, Ferguson DK, Zou C. *Lagerstroemia* (Lythraceae) pollen from the Miocene of eastern China. Grana. 2008;47(4):262–271. doi: 10.1080/00173130802457255

[94] Makbul S, Türkmen Z, Coskunçelebi K, Beyazoglu O. Anatomical and pollen characters in the genus *Epilobium* L. (Onagraceae) from Northeast Anatolia. Acta Biologica Cracoviensia. 2008;50(1):51–62.

[95] Thangaraja A, Ganesan V. Studies on the pollen biology of *Terminalia paniculata* Roth. (Combretaceae). African Journal of Plant Science. 2008;2(12):140–146.

[96] Chantaranothai P. Palynological studies in the family Melastomataceae from Thailand. Grana. 1997;36(3):146–159. doi: 10.1080/00173139709362602

[97] Carmo-Oliveira R, Morretes BLd. Stigmatic surface in the Vochysiaceae: reproductive and taxonomic implications. Acta Botanica Brasilica. 2009;23(3):780–785. doi: 10.1590/S0102-33062009000300018

[98] Grímsson F, Ferguson DK, Zetter R. Morphological trends in the fossil pollen of *Decodon* and the paleobiogeographic history of the genus. International Journal of Plant Sciences. 2012;173(3):297–317. doi: 10.1086/663968

[99] Grímsson F, Zetter R, Leng Q. Diverse fossil Onagraceae pollen from a Miocene palynoflora of north-east China: early steps in resolving the phytogeographic history of the family. Plant Systematics and Evolution. 2012;298(3):671–687. doi: 10.1007/s00606-011-0578-0

[100] Thornhill AH, Hope GS, Craven LA, Crisp MD. Pollen morphology of the Myrtaceae. Part 1: tribes Eucalypteae, Lophostemoneae, Syncarpieae, Xanthostemoneae and subfamily Psiloxyloideae. Australian Journal of Botany. 2012;60(3):165–199. doi: 10.1071/BT11174

[101] Thornhill AH, Hope GS, Craven LA, Crisp MD. Pollen morphology of the Myrtaceae. Part 2: tribes Backhousieae, Melaleuceae, Metrosidereae, Osbornieae and Syzygieae. Australian Journal of Botany. 2012;60(3):200–224.

[102] Thornhill AH, Wilson PG, Drudge J, Barrett MD, Hope GS, Craven LA, et al Pollen morphology of the Myrtaceae. Part 3: tribes Chamelaucieae, Leptospermeae and Lindsayomyrteae. Australian Journal of Botany. 2012;60(3):225–259. doi: 10.1071/BT11176

[103] Thornhill AH, Hope GS, Craven LA, Crisp MD. Pollen morphology of the Myrtaceae. Part 4: tribes Kanieae, Myrteae and Tristanieae. Australian Journal of Botany. 2012;60(3):260–289.

[104] Barth MO, Pinto da Luz CF. Pollen morphology of Vochysiaceae tree species in the State of Santa Catarina, southern Brazil. Revista de Biología Tropical. 2014;62(3):1209–1215. doi: 10.15517/rbt.v62i3.12323 25412545

[105] Schneider CA, Rasband WS, Eliceiri KW, et al NIH Image to ImageJ: 25 years of image analysis. Nature Methods. 2012;9(7):671–675. doi: 10.1038/nmeth.2089 22930834 10.1038/nmeth.2089 PMC5554542

[106] R Core Team. R: A Language and environment for statistical computing; 2015. Available from: https://www.R-project.org/.

[107] Hothorn T, Hornik K, Wiel MA, Zeileis A. Implementing a class of permutation tests: the coin package. Journal of Statistical Software. 2008;28(8):1–23. doi: 10.18637/jss.v028.i0827774042

[108] Hothorn T, Bretz F, Westfall P. Simultaneous inference in general parametric models. Biometrical Journal. 2008;50(3):346–363. doi: 10.1002/bimj.200810425 18481363 10.1002/bimj.200810425

[109] Mangiafico SS. An R companion for the handbook of biological statistics. Version 1.3.2. Available at rcompanion.org/rcompanion/; 2015.

[110] Tomizono S. boxplotdbl: Double box plot for two-axes correlation; 2013. Available from: https://CRAN.R-project.org/package=boxplotdbl.

[111] Katoh K, Standley DM. MAFFT multiple sequence alignment software version 7: improvements in performance and usability. Molecular Biology and Evolution. 2013;30(4):772–780. doi: 10.1093/molbev/mst010 23329690 10.1093/molbev/mst010 PMC3603318

[112] Stamatakis A. RAxML version 8: a tool for phylogenetic analysis and post-analysis of large phylogenies. Bioinformatics. 2014;30(9):1312–1313. doi: 10.1093/bioinformatics/btu033 24451623 10.1093/bioinformatics/btu033 PMC3998144

[113] Miller MA, Pfeiffer W, Schwartz T. Creating the CIPRES Science Gateway for inference of large phylogenetic trees. In: Proceedings of the Gateway Computing Environments Workshop (GCE) New Orleans, LA; 2010. p. 1–8.

[114] Smith SA, O’Meara BC. treePL: divergence time estimation using penalized likelihood for large phylogenies. Bioinformatics. 2012;28(20):2689–2690. doi: 10.1093/bioinformatics/bts492 22908216 10.1093/bioinformatics/bts492

[115] Boyle B, Hopkins N, Lu Z, Garay JAR, Mozzherin D, Rees T, et al The taxonomic name resolution service: an online tool for automated standardization of plant names. BMC Bioinformatics. 2013;14(1):1 doi: 10.1186/1471-2105-14-16 23324024 10.1186/1471-2105-14-16 PMC3554605

[116] Revell LJ. phytools: an R package for phylogenetic comparative biology (and other things). Methods in Ecology and Evolution. 2012;3(2):217–223. doi: 10.1111/j.2041-210X.2011.00169.x

[117] Ho LST, Ané C. A linear-time algorithm for Gaussian and non-Gaussian trait evolution models. Systematic Biology. 2014;63(3):397–408. doi: 10.1093/sysbio/syu005 24500037 10.1093/sysbio/syu005

[118] Hansen TF. Stabilizing selection and the comparative analysis of adaptation. Evolution. 1997;51(5):1341–1351. doi: 10.2307/2411186 28568616 10.1111/j.1558-5646.1997.tb01457.x

[119] Butler MA, King AA. Phylogenetic comparative analysis: a modeling approach for adaptive evolution. The American Naturalist. 2004;164(6):683–695. doi: 10.1086/426002 10.1086/426002 29641928

[120] Beaulieu JM, Jhwueng DC, Boettiger C, O’Meara BC. Modeling stabilizing selection: expanding the Ornstein—Uhlenbeck model of adaptive evolution. Evolution. 2012;66(8):2369–2383. doi: 10.1111/j.1558-5646.2012.01619.x 22834738 10.1111/j.1558-5646.2012.01619.x

[121] Cressler CE, Butler MA, King AA. Detecting adaptive evolution in phylogenetic comparative analysis using the Ornstein—Uhlenbeck model. Systematic biology. 2015;64(6):953–968. doi: 10.1093/sysbio/syv043 26115662 10.1093/sysbio/syv043

[122] Khabbazian M, Kriebel R, Rohe K, Ané C. Fast and accurate detection of evolutionary shifts in Ornstein-Uhlenbeck models. Methods in Ecology and Evolution. 2016; doi: 10.1111/2041-210X.12534

[123] Tibshirani R. Regression shrinkage and selection via the lasso. Journal of the Royal Statistical Society Series B (Methodological). 1996; p. 267–288.

[124] Ho LST, Ané C. Intrinsic inference difficulties for trait evolution with Ornstein-Uhlenbeck models. Methods in Ecology and Evolution. 2014;5(11):1133–1146. doi: 10.1111/2041-210X.12285

[125] Ejsmond MJ, Wrońska-Pilarek D, Ejsmond A, Dragosz-Kluska D, Karpińska-Kołaczek M, Kołaczek P, et al Does climate affect pollen morphology? Optimal size and shape of pollen grains under various desiccation intensity. Ecosphere. 2011;2(10):1–15. doi: 10.1890/ES11-00147.1

[126] Rind D. Latitudinal temperature gradients and climate change. Journal of Geophysical Research: Atmospheres. 1998;103(D6):5943–5971. doi: 10.1029/97JD03649

[127] Frenne P, Graae BJ, Rodríguez-Sánchez F, Kolb A, Chabrerie O, Decocq G, et al Latitudinal gradients as natural laboratories to infer species’ responses to temperature. Journal of Ecology. 2013;101(3):784–795. doi: 10.1111/1365-2745.12074

[128] Kerkhoff AJ, Moriarty PE, Weiser MD. The latitudinal species richness gradient in New World woody angiosperms is consistent with the tropical conservatism hypothesis. Proceedings of the National Academy of Sciences. 2014;111(22):8125–8130. doi: 10.1073/pnas.1308932111 10.1073/pnas.1308932111 PMC4050539 24847062

[129] Maldonado C, Molina CI, Zizka A, Persson C, Taylor CM, Albán J, et al Estimating species diversity and distribution in the era of Big Data: to what extent can we trust public databases? Global Ecology and Biogeography. 2015;24(8):973–984. doi: 10.1111/geb.12326 27656106 10.1111/geb.12326 PMC5012125

[130] Knight CA, Clancy RB, Götzenberger L, Dann L, Beaulieu JM. On the relationship between pollen size and genome size. Journal of Botany. 2010;2010:1–7.

[131] Beaulieu JM, Leitch IJ, Patel S, Pendharkar A, Knight CA. Genome size is a strong predictor of cell size and stomatal density in angiosperms. New Phytologist. 2008;179(4):975–986. doi: 10.1111/j.1469-8137.2008.02528.x 18564303 10.1111/j.1469-8137.2008.02528.x

[132] Bennett M, Leitch I. *Plant DNA C-values Database*; 2001. Available from: http://www.kew.org/cvalues/ [accessed September 2015].

[133] Narayan R. Discontinuous DNA variation in the evolution of plant species. Journal of Genetics. 1985;64(2–3):101–109. doi: 10.1007/BF02931138

[134] Narayan R. The role of genomic constraints upon evolutionary changes in genome size and chromosome organization. Annals of Botany. 1998;82(suppl 1):57–66. doi: 10.1006/anbo.1998.0752

[135] Pires JC, Hertweck KL. A renaissance of cytogenetics: studies in polyploidy and chromosomal evolution. Annals of the Missouri Botanical Garden. 2008;95(2):275–281. doi: 10.3417/2007176

[136] Edlund AF, Swanson R, Preuss D. Pollen and stigma structure and function: the role of diversity in pollination. The Plant Cell. 2004;16(suppl 1):S84–S97. doi: 10.1105/tpc.015800 15075396 10.1105/tpc.015800 PMC2643401

[137] Harder LD. Pollen-size comparisons among animal-pollinated angiosperms with different pollination characteristics. Biological Journal of the Linnean Society. 1998;64(4):513–525. doi: 10.1111/j.1095-8312.1998.tb00347.x

[138] Muller J. Form and function in angiosperm pollen. Annals of the Missouri Botanical Garden. 1979;66:593–632. doi: 10.2307/2398913

[139] Payne WW. Structure and function in angiosperm pollen wall evolution. Review of Palaeobotany and Palynology. 1981;35(1):39–59. doi: 10.1016/0034-6667(81)90013-0

[140] Katifori E, Alben S, Cerda E, Nelson DR, Dumais J. Foldable structures and the natural design of pollen grains. Proceedings of the National Academy of Sciences. 2010;107(17):7635–7639. doi: 10.1073/pnas.0911223107 10.1073/pnas.0911223107 PMC2867878 20404200

[141] Volkova OA, Severova EE, Polevova SV. Structural basis of harmomegathy: evidence from Boraginaceae pollen. Plant Systematics and Evolution. 2013;299(9):1769–1779. doi: 10.1007/s00606-013-0832-8

[142] Scotland RW, Barnes SH, Blackmore S. Harmomegathy in the Acanthaceae: a preliminary study of three pseudocolpate species using low temperature scanning electron microscopy. Grana. 1990;29(1):37–45. doi: 10.1080/00173139009429975

[143] Van Der Hamen RWJM, Van Heuven BJ. Evolutionary trends in the morphology and harmomegathy of the pollen of the genus *Guioa* (Sapindaceae-Cupanieae). Blumea. 1989;34(1):21–60.

[144] Muller J. Exine architecture and function in some Lythraceae and Sonneratiaceae. Review of Palaeobotany and Palynology. 1981;35(1):93–123. doi: 10.1016/0034-6667(81)90016-6

[145] Soltis DE, Smith SA, Cellinese N, Wurdack KJ, Tank DC, Brockington SF, et al Angiosperm phylogeny: 17 genes, 640 taxa. American Journal of Botany. 2011;98(4):704–730. doi: 10.3732/ajb.1000404 21613169 10.3732/ajb.1000404

[146] Sytsma KJ, Spalink D, Berger B. Calibrated chronograms, fossils, outgroup relationships, and root priors: re-examining the historical biogeography of Geraniales. Biological Journal of the Linnean Society. 2014;113(1):29–49. doi: 10.1111/bij.12297

[147] Palazzesi L, Gottschling M, Barreda V, Weigend M. First Miocene fossils of Vivianiaceae shed new light on phylogeny, divergence times, and historical biogeography of Geraniales. Biological Journal of the Linnean Society. 2012;107(1):67–85. doi: 10.1111/j.1095-8312.2012.01910.x

[148] Fiz O, Vargas P, Alarcón M, Aedo C, García JL, Aldasoro JJ. Phylogeny and historical biogeography of Geraniaceae in relation to climate changes and pollination ecology. Systematic Botany. 2008;33(2):326–342. doi: 10.1600/036364408784571482

[149] Ksepka DT, Benton MJ, Carrano MT, Gandolfo MA, Head JJ, Hermsen EJ, et al Synthesizing and databasing fossil calibrations: divergence dating and beyond. Biology Letters. 2011; doi: 10.1098/rsbl.2011.0356 10.1098/rsbl.2011.0356 PMC3210658 21525049

[150] Parham JF, Donoghue PC, Bell CJ, Calway TD, Head JJ, Holroyd PA, et al Best practices for justifying fossil calibrations. Systematic Biology. 2011; doi: 10.1093/sysbio/syr107 22105867 10.1093/sysbio/syr107 PMC3280042

[151] Sauquet H, Ho SY, Gandolfo MA, Jordan GJ, Wilf P, Cantrill DJ, et al Testing the impact of calibration on molecular divergence times using a fossil-rich group: the case of *Nothofagus* (Fagales). Systematic Biology. 2012;61(2):289–313. doi: 10.1093/sysbio/syr116 22201158 10.1093/sysbio/syr116

[152] Magallón S, Hilu KW, Quandt D. Land plant evolutionary timeline: gene effects are secondary to fossil constraints in relaxed clock estimation of age and substitution rates. American Journal of Botany. 2013;100(3):556–573. doi: 10.3732/ajb.1200416 23445823 10.3732/ajb.1200416

[153] Wilf P, Escapa IH. Green Web or megabiased clock? Plant fossils from Gondwanan Patagonia speak on evolutionary radiations. New Phytologist. 2015;207(2):283–290. doi: 10.1111/nph.13114 25441060 10.1111/nph.13114

[154] Thornhill AH, Ho SY, Külheim C, Crisp MD. Interpreting the modern distribution of Myrtaceae using a dated molecular phylogeny. Molecular Phylogenetics and Evolution. 2015;93:29–43. doi: 10.1016/j.ympev.2015.07.007 26211451 10.1016/j.ympev.2015.07.007

[155] Mayr G, Wilde V. Eocene fossil is earliest evidence of flower-visiting by birds. Biology Letters. 2014;10(5). doi: 10.1098/rsbl.2014.0223 24872461 10.1098/rsbl.2014.0223 PMC4046380

