# Supplementary figures and images for: A continuous morphological approach to study the evolution of pollen in a phylogenetic context: An example with the order Myrtales
Source: PLoS One. 2017 Dec 6;12(12):e0187228. doi: 10.1371/journal.pone.0187228 (PMC5718504; doi:10.1371/journal.pone.0187228)

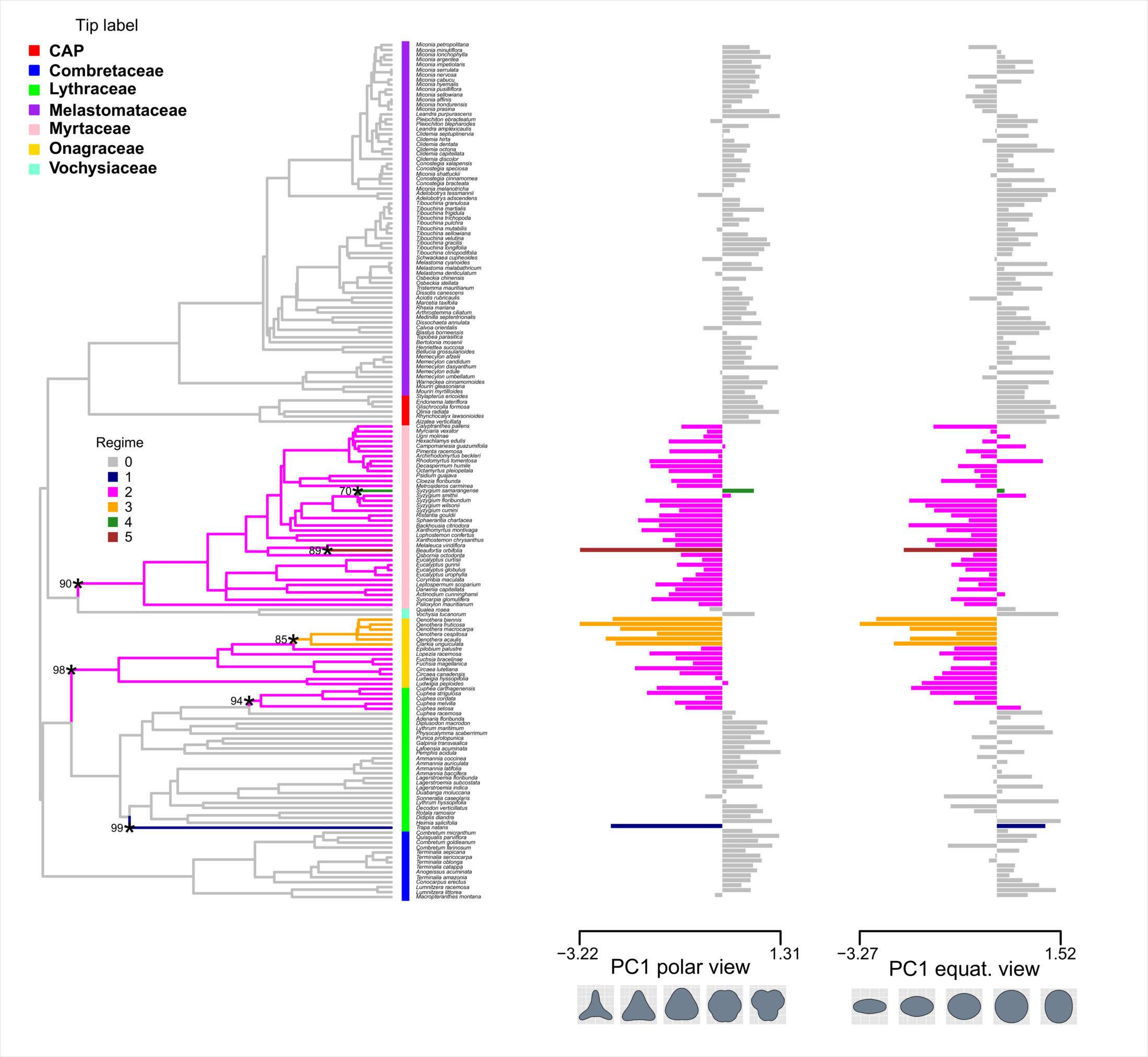

Supplement: S1 Fig — The color of the edges of the tree and the bars of the bar plot indicate the regime number of that clade. Asterisks highlight edges where shifts occurred and numbers at their side indicate bootstrap support for the corresponding shift. Only shifts with more than 50% bootstrap support are annotated. Bar plots next to the tree represent the trait values. (TIF) [file pone.0187228.s001.tif]

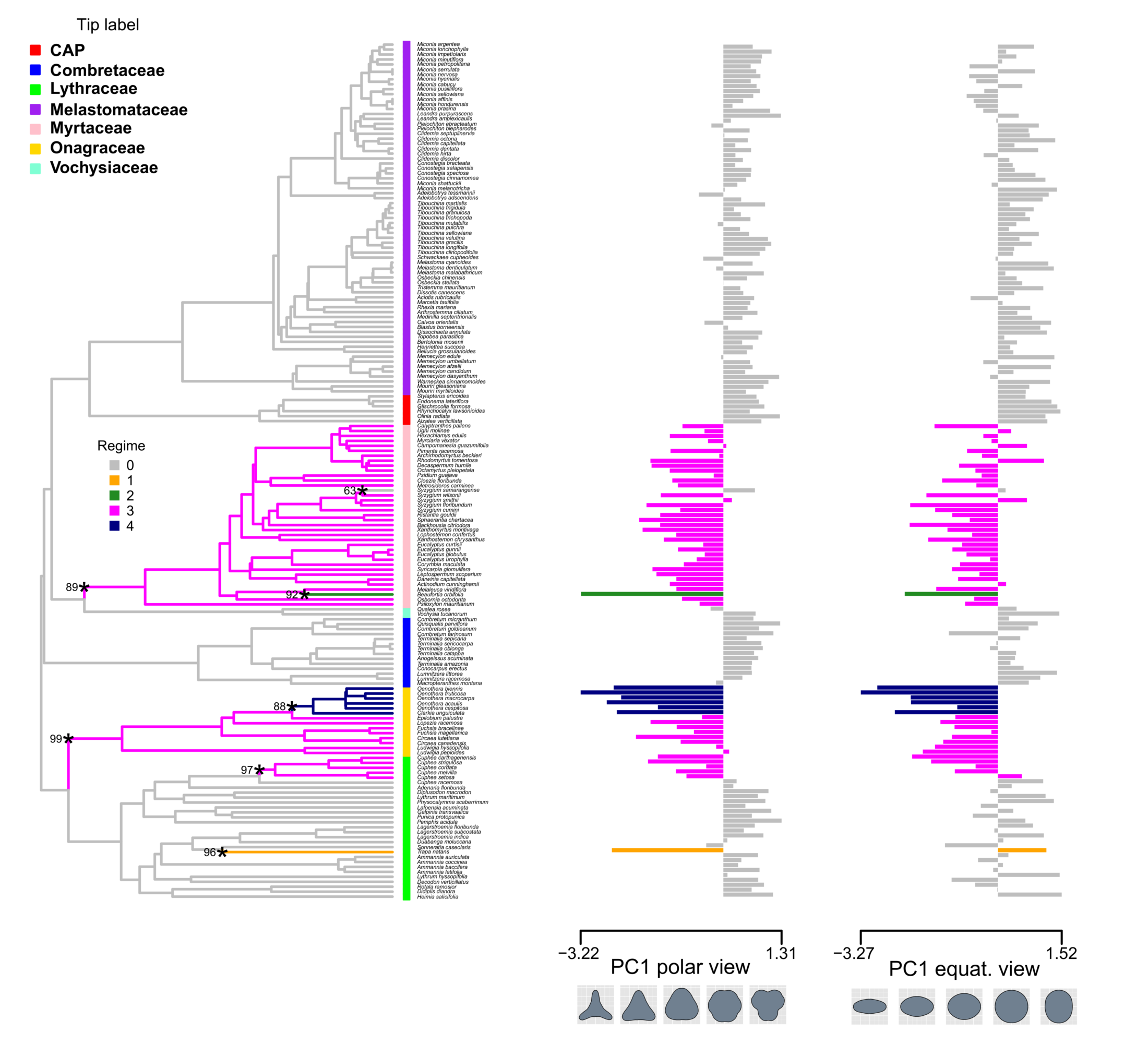

Supplement: S2 Fig — The color of the edges of the tree and the bars of the bar plot indicate the regime number of that clade. Asterisks highlight edges where shifts occurred and numbers at their side indicate bootstrap support for the corresponding shift. Only shifts with more than 50% bootstrap support are annotated. Bar plots next to the tree represent the trait values. (TIF) [file pone.0187228.s002.tif]

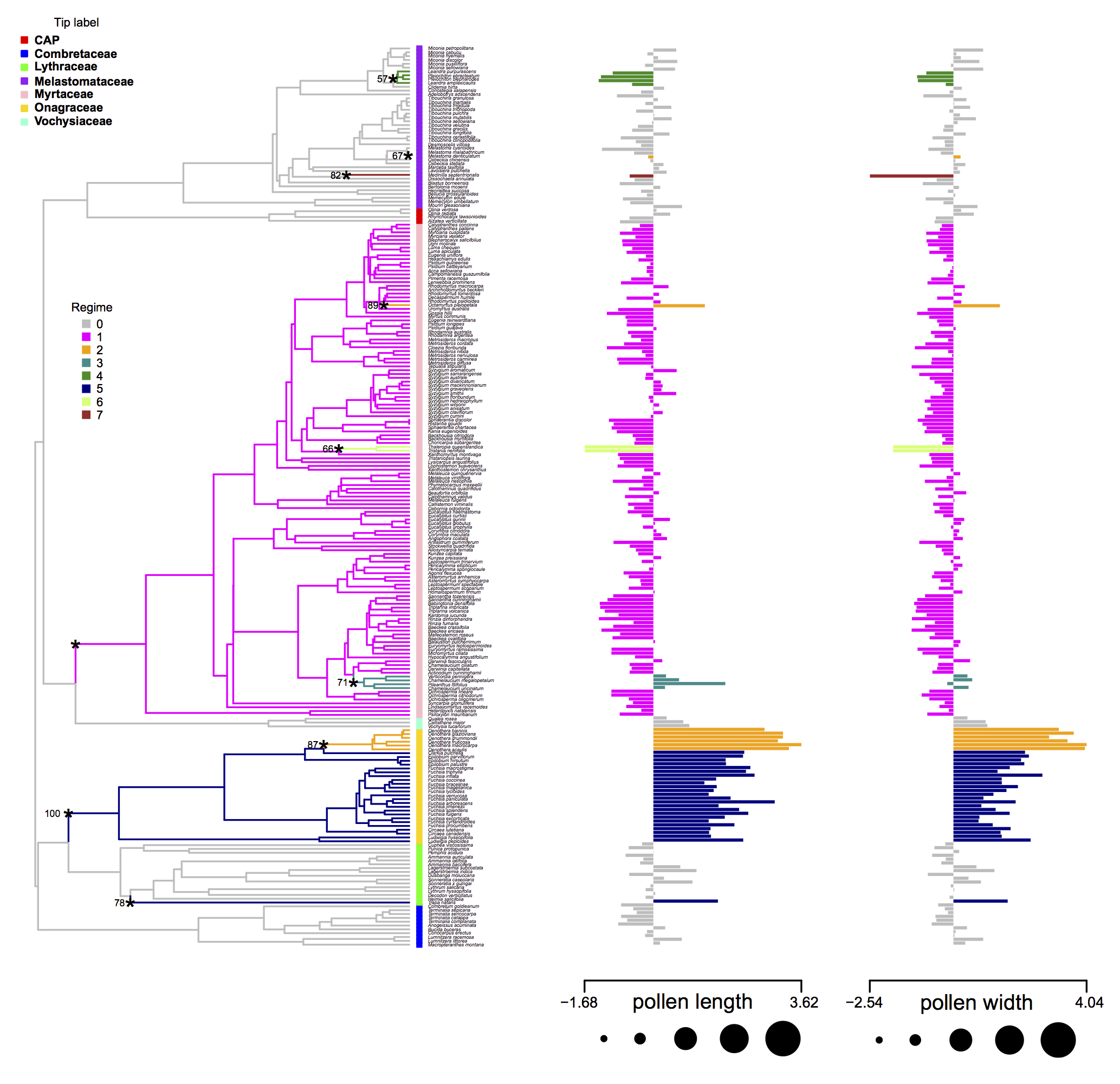

Supplement: S3 Fig — The color of the edges of the tree and the bars of the bar plot indicate the regime number of that clade. Asterisks highlight edges where shifts occurred and numbers at their side indicate bootstrap support for the corresponding shift. Only shifts with more than 50% bootstrap support are annotated. Bar plots next to the tree represent the trait values. (TIF) [file pone.0187228.s003.tif]

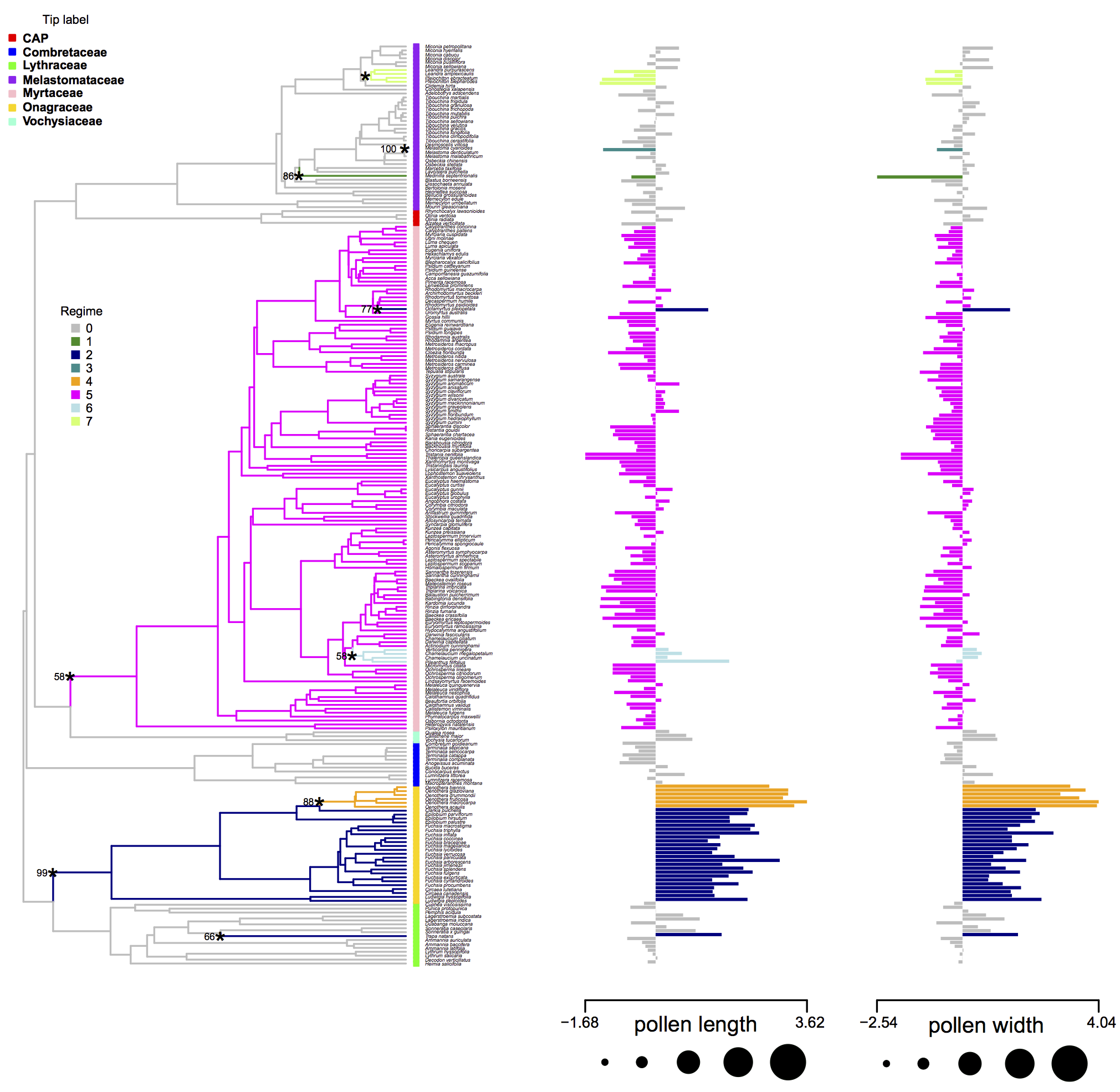

Supplement: S4 Fig — The color of the edges of the tree and the bars of the bar plot indicate the regime number of that clade. Asterisks highlight edges where shifts occurred and numbers at their side indicate bootstrap support for the corresponding shift. Only shifts with more than 50% bootstrap support are annotated. Bar plots next to the tree represent the trait values. (TIF) [file pone.0187228.s004.tif]

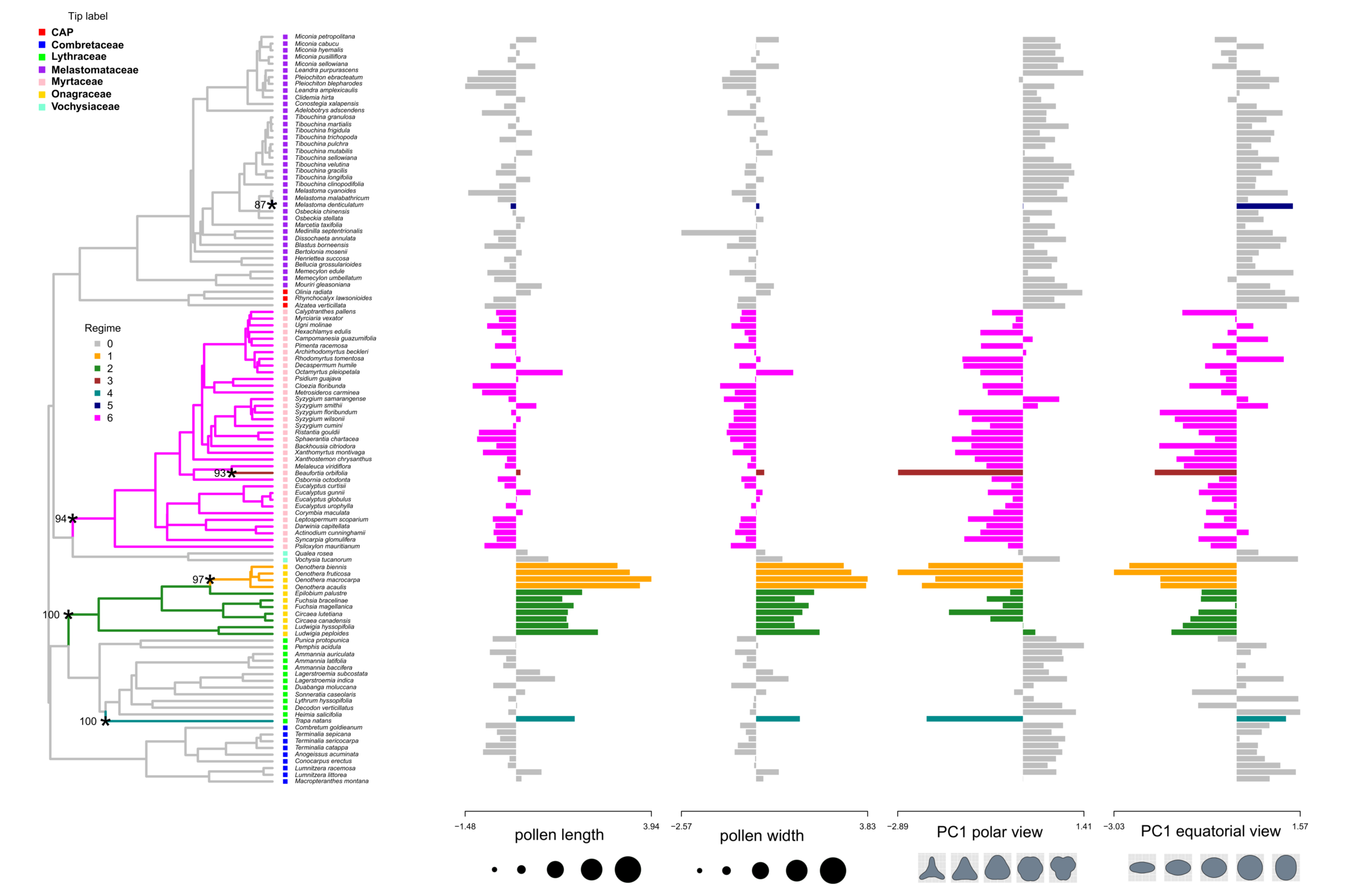

Supplement: S5 Fig — The color of the edges of the tree and the bars of the bar plot indicate the regime number of that clade. Asterisks highlight edges where shifts occurred and numbers at their side indicate bootstrap support for the corresponding shift. Only shifts with more than 50% bootstrap support are annotated. Bar plots next to the tree represent the trait values. (TIF) [file pone.0187228.s005.tif]

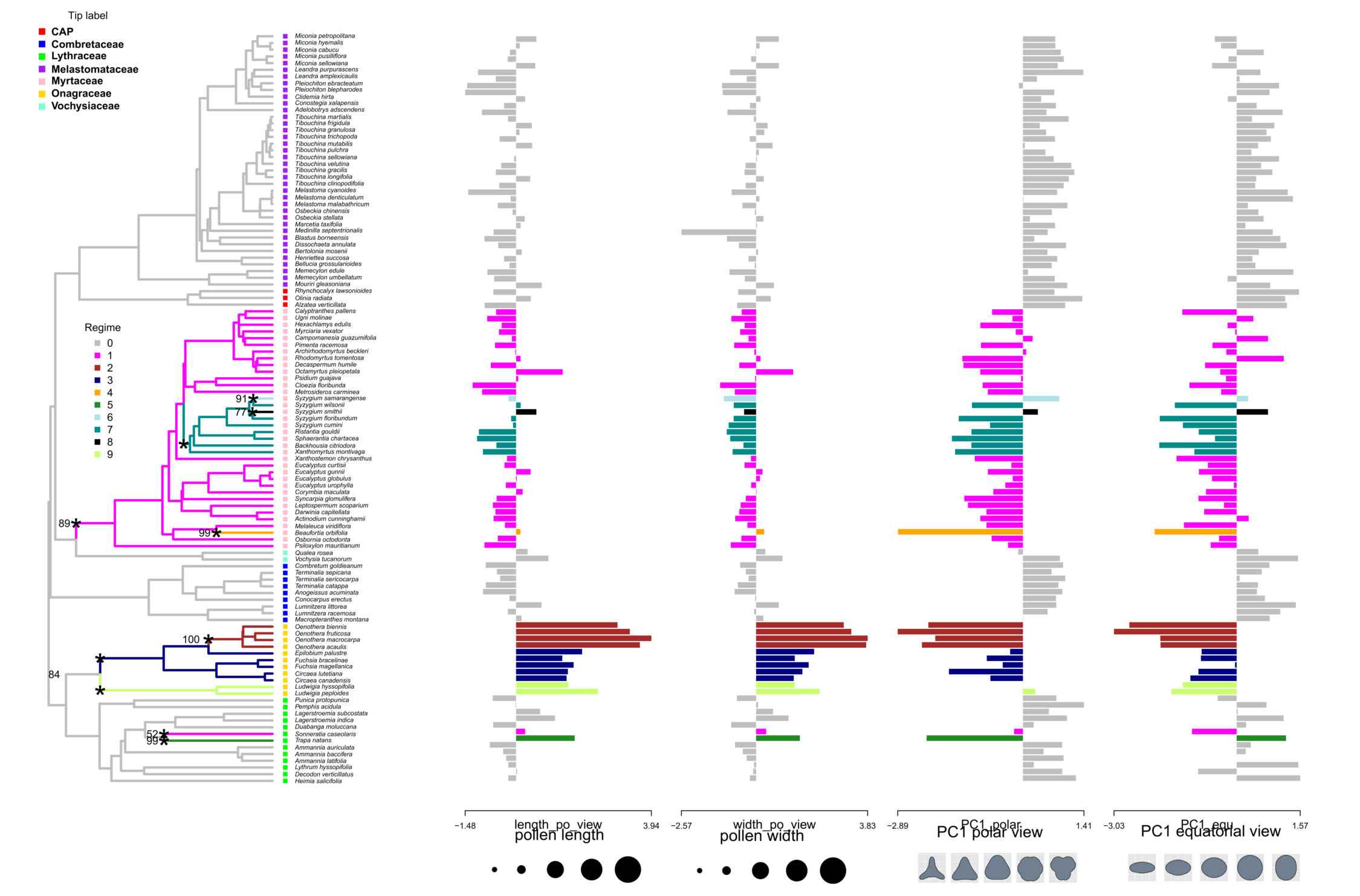

Supplement: S6 Fig — The color of the edges of the tree and the bars of the bar plot indicate the regime number of that clade. Asterisks highlight edges where shifts occurred and numbers at their side indicate bootstrap support for the corresponding shift. Only shifts with more than 50% bootstrap support are annotated. Bar plots next to the tree represent the trait values. (TIF) [file pone.0187228.s006.tif]
